# Distinct Roles for Laminin Globular Domains in Laminin α1 Chain Mediated Rescue of Murine Laminin α2 Chain Deficiency

**DOI:** 10.1371/journal.pone.0011549

**Published:** 2010-07-19

**Authors:** Kinga I. Gawlik, Mikael Åkerlund, Virginie Carmignac, Harri Elamaa, Madeleine Durbeej

**Affiliations:** Department of Experimental Medical Science, Muscle Biology Unit, University of Lund, Lund, Sweden; Hospital Vall d'Hebron, Spain

## Abstract

**Background:**

Laminin α2 chain mutations cause congenital muscular dystrophy with dysmyelination neuropathy (MDC1A). Previously, we demonstrated that laminin α1 chain ameliorates the disease in mice. Dystroglycan and integrins are major laminin receptors. Unlike laminin α2 chain, α1 chain binds the receptors by separate domains; laminin globular (LG) domains 4 and LG1-3, respectively. Thus, the laminin α1 chain is an excellent tool to distinguish between the roles of dystroglycan and integrins in the neuromuscular system.

**Methodology/Principal Findings:**

Here, we provide insights into the functions of laminin α1LG domains and the division of their roles in MDC1A pathogenesis and rescue. Overexpression of laminin α1 chain that lacks the dystroglycan binding LG4-5 domains in α2 chain deficient mice resulted in prolonged lifespan and improved health. Importantly, diaphragm and heart muscles were corrected, whereas limb muscles were dystrophic, indicating that different muscles have different requirements for LG4-5 domains. Furthermore, the regenerative capacity of the skeletal muscle did not depend on laminin α1LG4-5. However, this domain was crucial for preventing apoptosis in limb muscles, essential for myelination in peripheral nerve and important for basement membrane assembly.

**Conclusions/Significance:**

These results show that laminin α1LG domains and consequently their receptors have disparate functions in the neuromuscular system. Understanding these interactions could contribute to design and optimization of future medical treatment for MDC1A patients.

## Introduction

Congenital muscular dystrophy type 1A (MDC1A) is an autosomal recessive disorder caused by mutations in the gene encoding laminin (LM) α2 chain. The general clinical hallmarks of MDC1A include neonatal onset of muscle weakness, hypotonia often associated with joint contractures, inability to stand and walk, elevated levels of creatine kinase, white matter abnormalities and dysmyelination neuropathy. Histological changes of muscles comprise fiber size variability, massive degeneration and extensive connective tissue infiltration. Most patients die as teenagers since there is no treatment for this devastating disease [1]. Several mouse models for MDC1A exist (e.g. generated LMα2 chain mutants *dy^3K^/dy^3K^*and *dy^W^/dy^W^* and the spontaneous mutant mouse strain *dy/dy*) and they adequately mirror the human condition [2]–[4].

LMs are extracellular proteins formed by α, β and γ chains. Together with other extracellular matrix components LMs form specialized extracellular matrices called basement membranes [5]. LM-211 (composed of α2, β1 and γ1 chains) is the major LM isoform expressed in muscle and peripheral nerve. Through interaction with transmembrane receptors it regulates major functions of the neuromuscular system and provides structural support to muscle fibers [6]. In muscle, at least two distinct protein complexes are known to be the key receptors for LMα2 chain; dystroglycan and integrin α7β1. Their importance is underscored by the fact that absence of integrin α7 chain, as well as hypoglycosylation of α-dystroglycan cause various forms of congenital muscular dystrophy [7], [8]. Furthermore, different studies involving manipulation of the dystroglycan gene in mice revealed an important role for dystroglycan in skeletal muscle [9]–[11]. Several studies indicated that the function of integrin α7 subunit and dystroglycan, being a part of the dystrophin-glycoprotein complex, could overlap [12]–[14]. However, recent studies show that whereas both dystroglycan and integrin α7 chain contribute to force-production of muscles, only dystroglycan contributes to the preservation of sarcolemmal integrity [15].

LMα2 chain receptors present in peripheral nerve include dystroglycan, integrins α6β1, α7β1 and possibly integrin α6β4 [16], [17]. Dystroglycan, β1 and β4 integrin subunits have been shown to be important for different aspects of myelination and morphology of peripheral nerves, as revealed by conditional disruption of their genes in Schwann cells [18]–[20]. Thus, LM-211 is a central player linking these receptors and their functions in the neuromuscular system.

LMα1 chain also binds to dystroglycan, integrin α6β1 and integrin α7β1 (and perhaps integrin α6β4) [17], [21]–[24]. Yet, it is not expressed in the neuromuscular system [25]. We have previously explored the possibilities of paralogous gene therapy for MDC1A and demonstrated that LMα1 chain is an excellent substitute for LMα2 chain in murine muscle, peripheral nerve and testis [25]–[28]. Additionally, LMα2 chain deficiency leads to perturbed expression of integrin α7 subunit, and reduced expression of the core protein of α-dystroglycan (but not α-dystroglycan glycosylation), at the sarcolemma [29]–[31]. Notably, LMα1 chain overexpression restores integrin α7 chain expression, indicating that this receptor could be crucial for improvement of muscle function in dystrophic animals [32].

**Figure 1:**
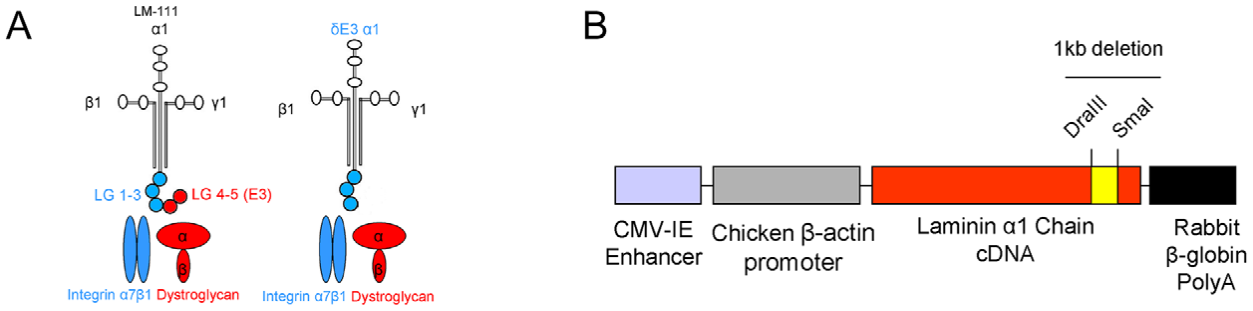
Generation of δE3LMα1 transgenic animals. (A) Scheme presenting LM-111 structure. Full-length LMα1 chain with LG1-5 domains and truncated LMα1 chain (δE3LMα1) with LG1-3 domains are marked together with their transmembrane receptors. (B) Schematic presentation of transgenic construct with denoted 1 kb deletion (LG4-5). Restriction sites used to engineer the construct are shown.

The LMα1 and α2 chains bind dystroglycan and integrins by distinct domains. The α1 chain binds dystroglycan via its C-terminal LG4 domain and integrin binding occurs via α1LG1-3 [33], [34]. This is different from LMα2 chain binding where there is considerable overlap in binding to dystroglycan and integrins. Both α2LG4-5 and α2LG1-3 bind dystroglycan, whereas only α2LG1-3 binds integrins [23], [35]. The LMα1 chain can thus be used more efficiently to distinguish between the roles of LM binding to dystroglycan and integrins in the neuromuscular system. Since LMα1 chain functions almost equally well as α2 chain in the neuromuscular system, we used this subunit in order to dissect the roles of the αLG domains and their receptors in MDC1A pathogenesis and rescue. Hence, we produced and characterized animals completely deficient in LMα2 chain, but instead overexpressing a truncated form of LMα1 chain (*dy^3K^*/δE3 mice) that lacks the dystroglycan binding site (LG4-5 domains at the C-terminus, also known as the E3 fragment), but retains the integrin binding site (LG1-3, see Fig. 1A) [33], [34].

## Materials and Methods

### Ethics statement

All mouse experimentation was approved by the local (Lund district) ethics committee (permit number M62-09). All mice were maintained in animal facilities according to animal care guidelines.

### Transgenic construct

Approximately 1 kb of the C-terminal part was removed from mouse full-length LMα1 chain cDNA to generate truncated cDNA (δE3LMα1). An in frame deletion between nucleotides 8248–9289 (corresponding to LG4-5 domains) was accomplished by DraIII-SmaI restriction cutting and fusion of an XhoI site with a BglII site. This DNA was cloned into the pCAGGS vector [25], containing a CMV enhancer and a β-actin promoter.

### Transgenic animals

Transgenic mice were generated by microinjections of transgene DNA into the pronucleus of fertilized single-cell C57BL/CBA embryos (Lund Transgenic Core Facility, Lund University, Sweden). Mice carrying δE3LMα1 chain DNA were identified by PCR as described previously [25]. Positive founders overexpressing truncated LMα1 chain in the neuromuscular system (lines No. 3 and 4) were further bred with dy^3K^/+ mice [2], followed by sib breeding to generate LMα2 chain deficient animals that express δE3LMα1 chain (dy^3K^/δE3 mice). Dy^3K^/dy^3K^ mice overexpressing full length LMα1 chain (dy^3K^LMα1 mice) were previously described [25]–[28]. Dy/dy mice used for heart studies were obtained from Jackson Laboratory.

### Exploratory locomotion and body and muscle weight analyses

Exploratory locomotion was examined in an open field test. A mouse was placed into a new cage and allowed to explore the cage for 5 min. The time that the mouse spent moving around was measured. For all experiments, 10-week-old dy^3K^/δE3 animals (n = 16) were compared with 10-week-old control mice (wild-type or dy^3K^/+) (n = 8) and 5-week-old dy^3K^/dy^3K^ mice (n = 6). For weight analysis dy^3K^/δE3, control mice and dy^3K^/dy^3K^ animals were sex- and age-matched (5-week-old) (n = 14, n = 3, n = 11, respectively, for females; n = 8, n = 4, n = 8, respectively, for males). Quadriceps and tibialis anterior muscles from 2-month-old wild-type (n = 3), dy^3K^/δE3 (n = 3) and 4-week-old dy^3K^/dy^3K^ mice (n = 4) were used to estimate the ratio of wet muscle weight to body weight. Muscles from both legs were weighed and average muscle mass was calculated. Unpaired t-test was used for statistical analysis.

### Creatine kinase activity

Blood was collected from the tail vein of 2-month-old control mice (wild-type or *dy^3K^/+*) (n = 10), *dy^3K^/*δE3 (n = 10) and 4-week-old *dy^3K^/dy^3K^* mice (n = 3) into EDTA-tubes and spun down two times for 5 minutes at 3500 rpm. CK_P_S_cobas method was used by Clinical Chemistry Laboratory at Skåne University Hospital to quantify enzyme activity in plasma. Unpaired t-test was used for statistical analyses.

### Histology and immunofluorescence microscopy

Skeletal muscle, heart, peripheral nerve and spinal roots cryosections (7 µm) from control (wild-type or *dy^3K^/+*), *dy^3K^/dy^3K^*, *dy/dy*, *dy^3K^*/δE3 and *dy^3K^*LMα1 mice were either stained with hematoxylin and eosin or subjected to immunofluorescence analysis using following antibodies: rat monoclonal mAb200 against LMα1LG4 domain [25], rabbit polyclonal 1057+ against LMα1 LN/LEa domain (N-terminus) (kindly provided by Dr. T. Sasaki) [36], rabbit polyclonal 1100+ against LMα4, (kindly provided by Dr. T. Sasaki), rabbit polyclonal 1113+ against LMα5 (kindly provided by Dr. T. Sasaki), rat monoclonal MTn15 against tenascin-C [25], rabbit polyclonal U31 against integrin α7B subunit (kindly provided by Dr. U. Mayer) [37], mouse monoclonal IIH6 against α-dystroglycan (Upstate Biotechnology), mouse monoclonal F1.652 against embryonic myosin heavy chain (Developmental Studies Hybridoma Bank, Iowa), rabbit polyclonal anti-collagen, type IV (Chemicon), mouse monoclonal 46 against caspase-3 (BD Transduction Laboratories). Mouse on mouse kit (Vector) was used for staining with embryonic myosin heavy chain according to manufacturer instructions. Tissues were fixed with 4% PFA at room temperature (for laminin, tenascin-C, embryonic myosin heavy chain, collagen-IV and caspase-3 stainings), or with acetone at −20°C (for integrin α7B) or with 8% formaldehyde, followed by methanol at −20°C (for α-dystroglycan). Sections were analyzed using a Zeiss Axioplan fluorescence microscope. Images were captured using an ORCA 1394 ER digital camera with Openlab 3 software. Images were prepared for publication using Adobe Photoshop software.

### Immunoblotting

For LM detection proteins were isolated from 100 mg of *dy^3K^*/δE3 and *dy^3K^*LMα1 muscles (3 mice from each group) by brief sonication in 1 mmol/L EDTA in TBS with 1∶25 dilution of protease inhibitors (Complete EDTA-free, Roche Diagnostics). For integrin detection proteins were extracted from 100 mg skeletal homogenized muscle powder of 3 wild-type and *dy^3K^*/δE3 mice in 1% Triton X-100, 50 mM Tris-HCl, pH 7,4; 1 mM CaCl_2_, 1 mM MgCl_2_ and 1∶25 dilution of Protease Cocktail (Complete EDTA-free, Roche Diagnostics). Samples were incubated for 1 hour and spun down at 4°C. The supernatants were collected and the protein concentration was determined using BCA assay (Pierce). Dystroglycan was isolated using agarose bound wheat germ agglutinin (Vector) and N-acetyl-D-glucosamine (Sigma) as described before [32]. Lysates containing LM, integrin and dystroglycan were separated on 5% or 8% polyacrylamide-SDS gels under reducing or non-reducing conditions. EHS LM (Invitrogen) was used as a control for LM blotting. Proteins were transferred to nitrocellulose membranes (Amersham). Membranes were blocked for 1 hour in 5% non-fat dry milk in 1xTBS with 0.02% Tween-20 and incubated overnight at 4°C with a rabbit polyclonal antibody detecting LMα1LG3 domain (kindly provided by Dr. T. Sasaki); rabbit polyclonal antibody against integrin α7B (kindly provided by Dr. U. Mayer); rabbit polyclonal antibody against β-dystroglycan [25] and mouse monoclonal antibody IIH6 against α-dystroglycan. Detection was performed with ECL kit (Amersham). Expression of LMα1 chain, integrin α7B subunit, α- and β-dystroglycan was normalized to α-actinin expression (detected with mouse monoclonal antibody EA-53, Sigma). Band intensity was measured using ImageJ software. Unpaired t-test was used for statistical analyses.

### Quantification of fiber size distribution, central nucleation and fiber number

Diaphragm and limb muscles from at least 3 animals from each group (4–6-week-old wild-type, *dy^3K^/dy^3K^* and *dy^3K^*/δE3 mice) were analyzed. Minimal Feret's diameter was measured [38] for at least 2600 fibers for each group. The same number of fibers was used for quantification of fibers with centrally located nuclei. An additional group of 4–6-month-old *dy^3K^*/δE3 animals was included for quantification of diaphragm fibers. Fibers from quadriceps muscle from 4–6-week-old wild-type (n = 3), *dy^3K^/dy^3K^* (n = 3) and *dy^3K^*/δE3 mice (n = 3) were counted within a square of 64×10^6^ pixels^2^. Unpaired t-test was used for statistical analysis.

### Treadmill exercise and Evans blue dye injection

Dy^3K^/δE3 mice (n = 4) were exercised for 30 min on a treadmill Exer 6M (Columbus Instruments) at a downhill angle of 15°. During the first 2 min the speed was gradually increased from 7 m/min up to 14–16 m/min. Within 30 min after completed exercise the mice were injected i.p. with Evans blue dye (EBD) (Sigma Aldrich) dissolved in sterile saline (concentration: 0.5 mg EBD/0.05 ml saline; amount: 50 µl per 10 g body weight). After approximately 24 h, muscles were collected and quickly frozen in liquid nitrogen. Unexercised mice were injected with EBD and used as controls. Muscle cryosections (8 µm) were fixed in ice-cold acetone at −20°C for 10 min, washed and mounted with FluorSave (Calbiochem). By fluorescence microscopy analysis, EBD uptake into muscle fibers was visualized by red emission.

### Cardiotoxin injections

Tibialis anterior muscles from 6 control (wild-type or *dy^3K^/+)*, 6 *dy^3K^/dy^3K^* and 6 *dy^3K^*/δE3 mice were injected with cardiotoxin (10 µmol/L in saline). Control and *dy^3K^*/δE3 mice were 2–3-month-old. *Dy^3K^/dy^3K^* mice were 3-week-old. Three mice from each group were sacrificed 4 days after injection and the other 3 after 11 days. Both injected and contralateral uninjected tibialis anterior muscles were collected and analyzed.

### Electron microscopy and toluidine blue staining

Quadriceps femoris muscles, heart, diaphragm, sciatic nerves and spinal roots from wild-type, *dy^3K^/dy^3K^* and *dy^3K^*/δE3 mice were fixed for 2 hours with 1.5% glutaraldehyde/1.5% paraformaldehyde, rinsed in Sörensen's phosphate buffer, post fixed in 1% OsO_4_ and then embedded in Epon. Ultra thin sections were stained with uranyl acetate and lead citrate. Specimens were examined by transmission electron microscopy (Philips CM 10). Three to 4 animals from each group were analyzed.

## Results

### Generation of *dy^3K^/dy^3K^* mice overexpressing δE3LMα1 chain

**Figure 2:**
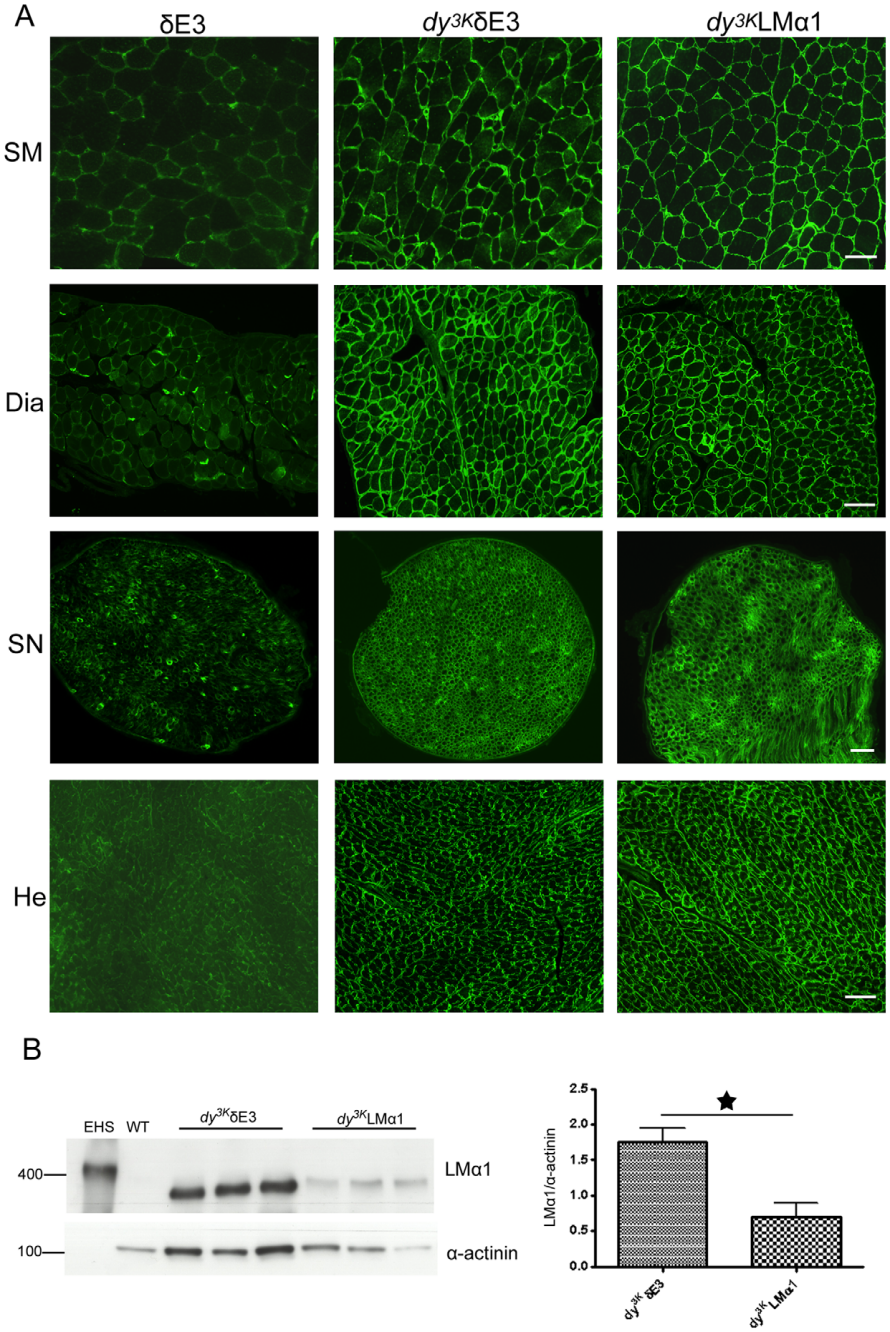
Comparison of expression levels of LMα1 chain between δE3 transgenic mice, *dy^3K^*/δE3 and *dy^3K^*LMα1 mice. (A) Truncated LMα1 chain is upregulated in skeletal muscle (SM), diaphragm (Dia), peripheral nerve (SN) and heart (He) in *dy^3K^*/δE3 mice compared to δE3 mice expressing LMα2 chain. It reaches the levels of full-length LMα1 chain expression in *dy^3K^*LMα1 mice. Three animals from each group were analyzed. Bars, 50 µm. (B) Immunoblotting of tissue extracts from wild-type, *dy^3K^*/δE3 and *dy^3K^*LMα1 skeletal muscle and EHS extract with a rabbit polyclonal antibody against LMα1LG3 domain. Quantification of signals revealed that there is approximately 2.5-fold more of truncated LMα1 chain in *dy^3K^*/δE3 muscles compared to full-length LMα1 chain in *dy^3K^*LMα1 muscles (p = 0.0194). Results are shown as means ± SEM. The shift in molecular weight of truncated (350 kDa) vs. full-length (400 kDa) LMα1 chain became apparent after running the samples for a longer time (data not shown).

We have generated mice overexpressing LMα1 chain devoid of LG4-5 domains (comprising the E3 fragment) under the control of a CMV enhancer and β-actin promoter (Fig. 1A and B) (δE3 mice), Mice overexpressing δE3LMα1 in skeletal muscle, peripheral nerve and heart were maintained (transgenic lines No. 3 and 4) (Figure S1, see also Fig. 2). The expression of truncated LMα1 chain was detected using antibodies against the N-terminal domains of LMα1 chain and the LG4 domain, respectively. Immunofluorescence staining with the antibody directed against N-terminal domains of LMα1 chain demonstrated patchy expression of truncated LMα1 chain in basement membranes of skeletal and cardiac muscle, and in endoneurium and perineurium of sciatic nerve of δE3 transgenic mice (Figure S1). No staining was detected with the antibody directed towards LG4 domain, indicating the overexpression of truncated LMα1 chain. Staining with both antibodies was detected in LMα1TG mice overexpressing full-length LMα1 chain (Figure S1) (described in 25) and it indicated a higher level and more homogeneous expression of LMα1 chain in these animals. Notably, overexpression of truncated LMα1 chain in mice revealed no discernible pathological phenotypes.

Next, δE3 mice from line 3 and 4 were further mated with mice carrying the mutation in Lama2 gene (*dy^3K^*/+), to create mice that are devoid of LMα2 chain but instead overexpress δE3LMα1 chain (*dy^3K^*/δE3 mice).

### Expression of truncated LMα1 chain is upregulated upon LMα2 chain deficiency

We analyzed the expression of δE3LMα1 chain in *dy^3K^*/δE3 mice in a similar manner as in δE3 mice (only the staining with the antibody against N-terminal domains is shown). Interestingly, upon LMα2 chain deficiency the truncated LMα1 chain was upregulated in all examined tissues (skeletal muscle, diaphragm, heart, peripheral nerve) compared to δE3 mice (Fig. 2A). Also, the expression levels seemed to reach those detected in *dy^3K^*LMα1 mice overexpressing full-length LMα1 chain. We also noted intracellular staining of truncated LMα1 chain in skeletal muscle (Fig. 2A). Western blot analyses with an antibody against LMα1LG3 domain revealed even higher expression (approximately 2.5-fold) of δE3LMα1 chain in *dy^3K^*/δE3 muscles compared to full-length LMα1 chain in *dy^3K^*LMα1 muscles (Fig. 2B). Therefore, we can rule out the possibility that the observed phenotype of *dy^3K^*/δE3 mice described below is due to insufficient expression of truncated LMα1 chain. Also, it is clear that the regulatory mechanisms involved in LMα1 chain transgene expression are complex. We also assessed the expression of LMα4 and α5 chains. We and others have previously shown that expression of these two LM chains is upregulated in LMα2 chain deficient basement membranes [25], [39] (see also Figure S2). In *dy^3K^*/δE3 mice, the muscle basement membrane expression of LMα4 and α5 chains was very similar to that of *dy^3K^/dy^3K^* mice (Figure S2). Hence, we suggest that the compensatory increase of LMα4 and LMα5 chains has no beneficial effects in *dy^3K^*/δE3 muscles (which are analyzed in detail in the next paragraphs).

### Expression of integrin α7B and dystroglycan in *dy^3K^/*δE3 tissues

**Figure 3:**
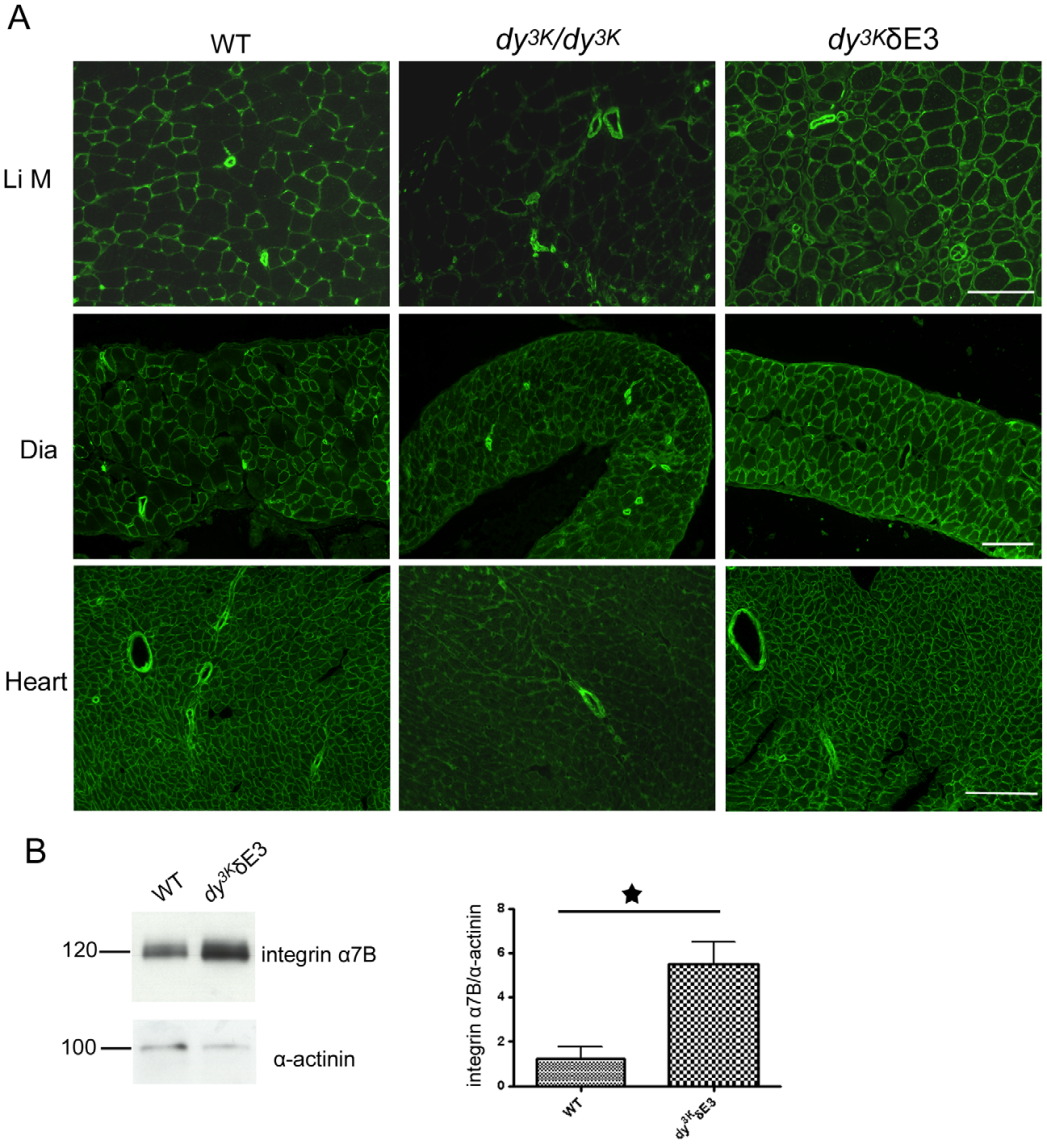
Restoration and upregulation of integrin α7B subunit in *dy^3K^*/δE3 muscles. (A) Cross-sections of limb muscle (Li M), diaphragm (Dia) and heart from wild-type, *dy^3K^/dy^3K^* and *dy^3K^*/δE3 mice were stained with antibodies against integrin α7B. Bars, 50 µm. (B) Immunoblotting of total protein lysates from wild-type and *dy^3K^*/δE3 skeletal muscle and quantitative measurement of integrin α7B expression. There is approximately 4.5-fold more integrin α7B in *dy^3K^*/δE3 muscle (p = 0.0231). Results are shown as means ± SEM.

We next evaluated the expression of integrin α7B and dystroglycan in *dy^3K^/*δE3 muscles. Expression of integrin α7B is reduced at the sarcolemma of *dy^3K^/dy^3K^* limb and heart muscle but to a lesser extent in *dy^3K^/dy^3K^* diaphragm (Fig. 3A). Notably, the expression of integrin α7B subunit was restored in *dy^3K^/*δE3 limb, diaphragm and heart muscle (Fig. 3A). Similarly, also full-length LMα1 chain reconstituted integrin α7B chain at LMα2 chain deficient sarcolemma [32]. We further detected an approximately 4.5-fold upregulation of integrin α7B in *dy^3K^/*δE3 skeletal muscle by immunoblotting experiments (Fig. 3B).

**Figure 4:**
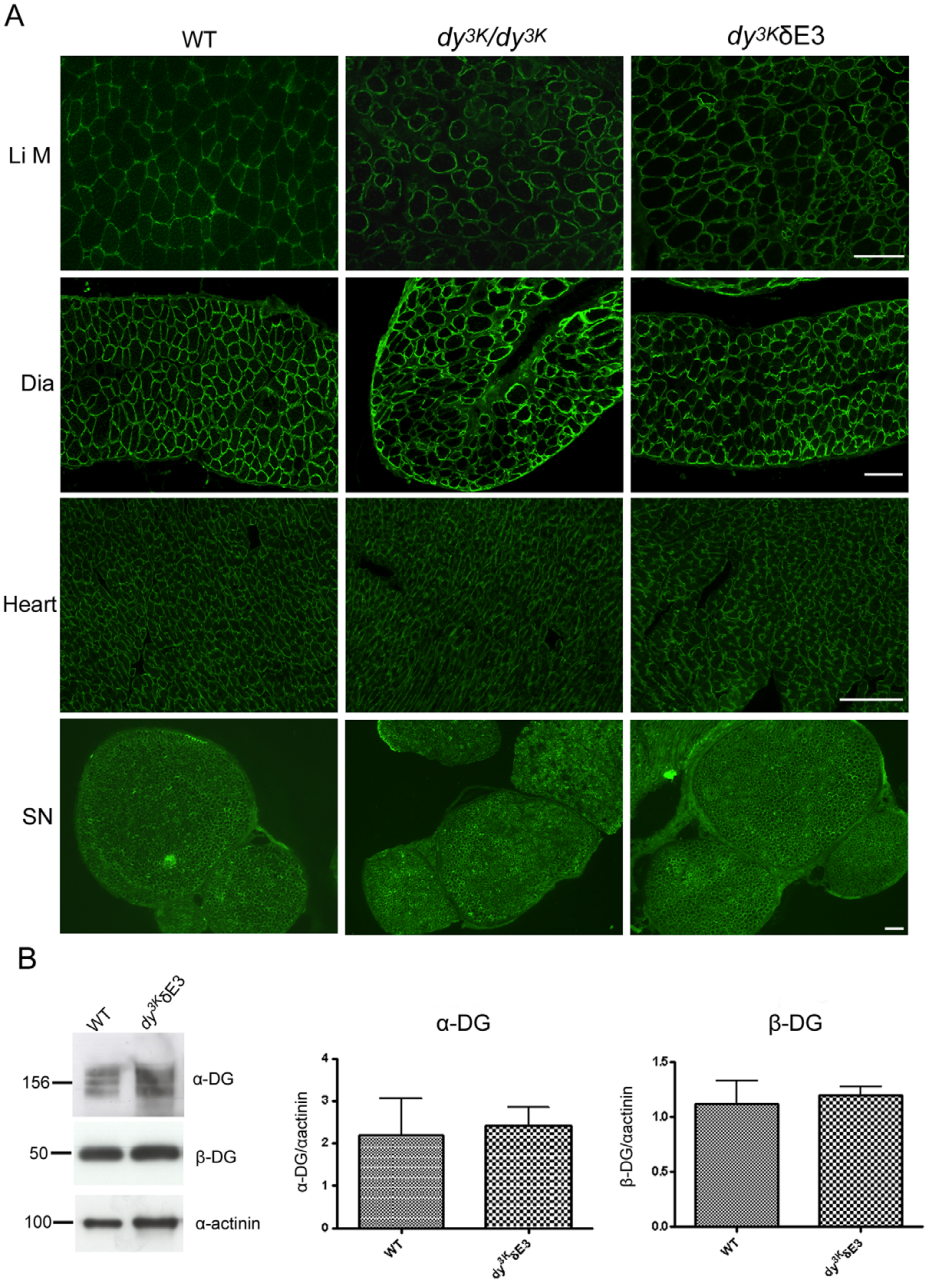
Normal expression of dystroglycans in *dy^3K^*/δE3 muscles. (A) Cross-sections of limb muscle (Li M), diaphragm (Dia), heart and sciatic nerve (SN) from wild-type, *dy^3K^/dy^3K^* and *dy^3K^*/δE3 mice were stained with antibody IIH6 against α-dystroglycan. Bars, 50 µm. (B) Immunoblotting of glycoprotein preparations from wild-type and *dy^3K^*/δE3 skeletal muscle and quantitative measurement of α- and β-dystroglycan expression. Results are shown as means ± SEM. No significant difference in expression of α- and β-dystroglycan was noted between wild-type and *dy^3K^*/δE3 muscle (p = 0.8200 and p = 0.7527, respectively).

LMα2 chain deficiency does not significantly alter α-dystroglycan glycosylation and β-dystroglycan expression at the sarcolemma [32], probably because other ligands (e.g. perlecan) are still present. By immunofluorescence analyses, expression of α-dystroglycan also appeared normal in *dy^3K^/*δE3 limb, diaphragm and heart muscle and in sciatic nerve (Fig. 4A). In addition, we quantified expression of α- and β-dystroglycan and they remained the same in *dy^3K^/*δE3 vs. control skeletal muscle (Fig. 4B).

All in all, these results suggest that integrin α7B is increased, whereas dystroglycans appear normally expressed in *dy^3K^/*δE3 muscles.

### *Dy^3K^/dy^3K^* mice with δE3LMα1 transgene have improved overall health

**Figure 5:**
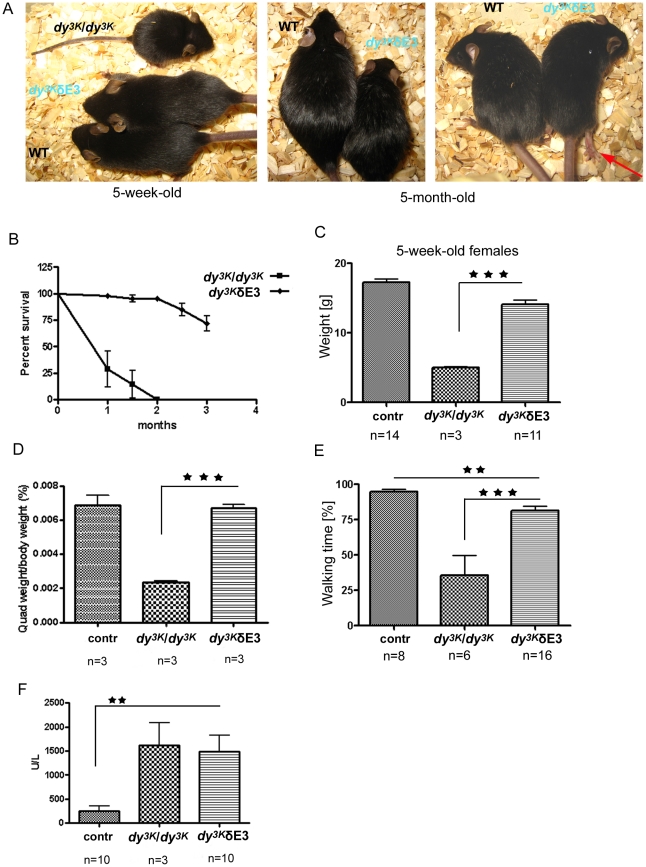
Overall phenotype of *dy^3K^*/δE3 mice. (A) 5-week-old *dy^3K^*/δE3 mice often have normal size, remain alert and lively with good muscle tone. A wild-type and a *dy^3K^/dy^3K^* littermate are shown for comparison. With age the difference between the body size of normal and *dy^3K^*/δE3 mice becomes more evident (middle panel). However some *dy^3K^*/δE3 animals (right panel) remain undistinguishable from littermates at older age. Nevertheless, all *dy^3K^*/δE3 mice develop peripheral neuropathy (indicated by arrow). (B) Survival curves of *dy^3K^/dy^3K^* (n = 8) and *dy^3K^*/δE3 mice (n = 44) up to 3 months of age. Curves remain significantly different from each other (p<0.0001). Around 75% of *dy^3K^*/δE3 mice live at least up to 3 months of age. (C) Whole body weights of 5-week-old female control, *dy^3K^/dy^3K^* and *dy^3K^*/δE3 mice. Body mass is partially recovered in female *dy^3K^*/δE3 mice. They weigh significantly more than *dy^3K^/dy^3K^* mice (p<0.0001), but significantly less than control animals p<0.0003). (D) Proportion (in percentage) of the wet weight of quadriceps muscle to the body weight in control, *dy^3K^/dy^3K^* and *dy^3K^*/δE3 mice. Compared to control mice, the ratio is normal in *dy^3K^*/δE3 (p = 0.8001) but significantly reduced in *dy^3K^/dy^3K^* mice (p = 0.0003). (E) Exploratory locomotion of 10-week-old control and *dy^3K^*/δE3 mice and 5-week-old *dy^3K^/dy^3K^* mice. *Dy^3K^*/δE3 mice are significantly more active than *dy^3K^/dy^3K^* mice (p<0.0001) and less active than control mice (p = 0.0099). (F) Serum creatine kinase (CK) activity in control, *dy^3K^/dy^3K^* and *dy^3K^*/δE3 mice. There is no difference in CK activity between *dy^3K^/dy^3K^* and *dy^3K^*/δE3 mice, but *dy^3K^*/δE3 remain significantly different from control mice (p = 0.0022) Each bar represents the mean ± SEM.

*Dy^3K^/dy^3K^* mice completely deficient in LMα2 chain were previously described [2]. Briefly, these animals suffer from severe muscle wasting, growth retardation, peripheral neuropathy and die approximately 3–5 weeks after birth. As shown in Fig. 5, the overall health of *dy^3K^*/δE3 mice was improved compared to *dy^3K^/dy^3K^* mice. First, *dy^3K^*/δE3 mice live longer. As demonstrated by the survival curve, approximately 75% of *dy^3K^*/δE3 animals survive up to 3 months (Fig. 5B). Further estimation of *dy^3K^*/δE3 survival encountered obstacles. Due to hindleg paralysis, several of them were sacrificed according to the guidelines of the ethical permit. Nevertheless, many *dy^3K^*/δE3 mice survive much longer than 3 months. Our oldest animals died one year after birth.

Second, *dy^3K^*/δE3 animals are bigger than *dy^3K^/dy^3K^* mice. At 2 weeks of age, *dy^3K^/dy^3K^* mice can be identified due to their growth retardation whereas *dy^3K^*/δE3 mice appeared outwardly normal (data not shown). Furthermore, the majority of *dy^3K^*/δE3 animals at 5 weeks of age can not be distinguished from normal littermates (Fig. 5A). Weight gain for female and male *dy^3K^/dy^3K^* mice was greatly delayed in 5-week-old mice whereas the weight gain for female and male *dy^3K^*/δE3 mice was significantly increased compared to *dy^3K^/dy^3K^* mice (Fig. 5C and data not shown). However, *dy^3K^*/δE3 mice weigh significantly less than normal littermates (Fig. 5C and data not shown). Beginning from 5 weeks of age, the difference in overall phenotype between most of *dy^3K^*/δE3 and wild-type mice became more evident. Many *dy^3K^*/δE3 animals are visibly smaller than control littermates (Fig. 5A, middle panel). However, some of the older *dy^3K^*/δE3 animals look outwardly normal and are almost indistinguishable from their littermates (Fig. 5A, left panel). Also, the ratio of quadriceps and tibialis anterior wet weight per body weight was similar in control and *dy^3K^*/δE3 mice, whereas the ratio was significantly reduced in *dy^3K^/dy^3K^* mice (Fig. 5D and data not shown). Hence, muscle mass was maintained in proportion to the body size in *dy^3K^*/δE3 mice. Nevertheless, most of *dy^3K^*/δE3 mice display severe peripheral nerve abnormalities, as demonstrated by temporary hindleg paralysis (either one or occasionally two limbs) (Fig. 5A, arrow). When lifted by the tail, they retract their hindlimbs toward the body. Still, *dy^3K^*/δE3 mice perform much better in the locomotion activity test compared to *dy^3K^/dy^3K^* animals (Fig. 5E), indicating that muscle function is largely preserved. Yet, *dy^3K^*/δE3 mice move significantly less than control mice and this is supposedly due to the temporary paralysis (Fig. 5E). Finally, we noted that serum kinase activity was significantly elevated in *dy^3K^*/δE3 mice (Fig. 5F), indicating that muscles may be dystrophic, despite improved general health.

In summary, survival during the first months of life and other features of the overall phenotype of *dy^3K^*/δE3 mice are not greatly dependent on LMα1LG4-5.

### ΔE3LMα1 transgene reduces the dystrophic pathology of skeletal muscles and significantly prevents dystrophic changes in diaphragm and heart

**Figure 6:**
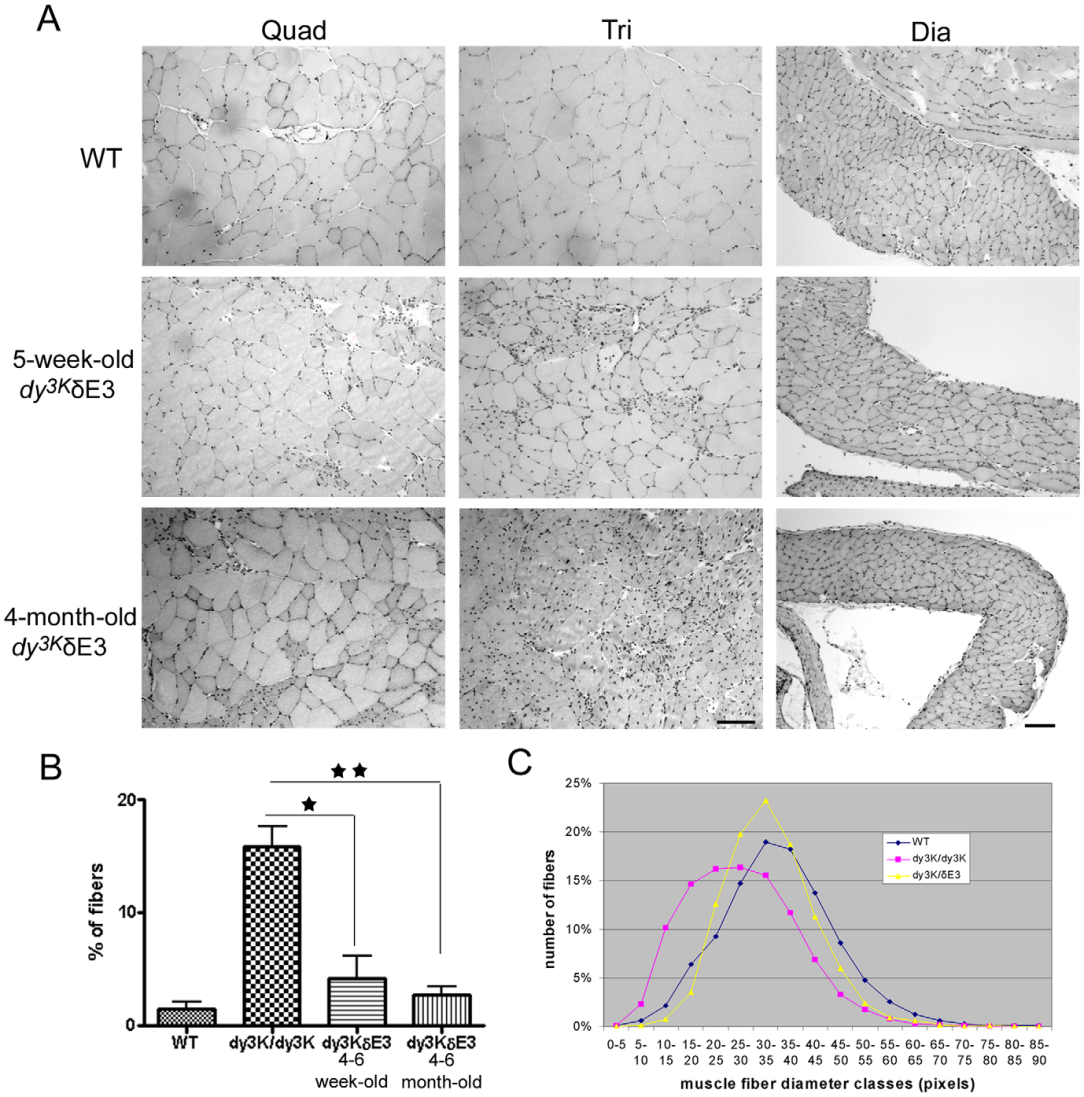
Analyses of muscle morphology and properties. (A) Hematoxylin and eosin staining of quadriceps femoris (Quad), triceps brachii (Tri) and diaphragm (Dia) muscles from 5-week-old and 4-month-old wild-type and *dy^3K^*/δE3 mice. Myopathic changes with groups of centrally nucleated muscle fibers were detected in quadriceps, and to larger extent in triceps of both 5-week-old and 4-month-old *dy^3K^*/δE3 mice. Central nucleation was not evident in diaphragm. Connective tissue infiltration was largely prevented in all muscle types. Three animals from each group were analyzed. (B) Quantification of central nucleation in 4–6-week-old wild-type, *dy^3K^/dy^3K^*, *dy^3K^*/δE3 and 4–6-month-old *dy^3K^*/δE3 diaphragm. The number of fibers with centrally located nuclei is not significantly different between wild-type and young or wild-type and old *dy^3K^*/δE3 diaphragm muscles (p = 0.2163 and p = 0.2707, respectively), whereas the number of regenerating fibers is significantly higher in *dy^3K^/dy^3K^* diaphragm compared to young and old *dy^3K^*/δE3 mice (p = 0.0255 and p = 0.0026). Each bar represents the mean ± SEM (p<0.05). At least 3 animals were analyzed. (C) Fiber size distribution in 4–6 week-old wild-type, *dy^3K^/dy^3K^*, *dy^3K^*/δE3 diaphragms. The *dy^3K^/dy^3K^* diaphragm fibers are smaller than *dy^3K^*/δE3 diaphragm fibers. Bars, 50 µm.

We next examined the morphology of *dy^3K^*/δE3 skeletal and heart muscle. When isolating skeletal muscles from *dy^3K^*/δE3 mice (5-week-old and 4-month-old and older), it could be macroscopically seen that muscles were only modestly wasted (see also Fig. 5D). However, histological analyses of muscle revealed vast regeneration of muscle fibers in limb muscles, demonstrated by the presence of small fibers with centrally located nuclei (Fig. 6A). Approximately 35% and 25% of 4–6-week-old *dy^3K^*/δE3 quadriceps and triceps muscle fibers, respectively, contained centrally located nuclei and the numbers of centrally nucleated fibers did not differ significantly from *dy^3K^/dy^3K^* muscles (data not shown). The number of fibers in randomly selected areas was similar in wild-type and *dy^3K^/*δE3 quadriceps muscle, but with a tendency of more fibers in *dy^3K^/*δE3 mice (probably due to the presence of small regenerating fibers). Interestingly, a similar number of fibers was also noted in *dy^3K^/dy^3K^* quadriceps muscle (Figure S3). However, average fiber diameter is smaller (data not shown) and instead muscle contains fibrotic tissue (see Figure 8A). The number of fibers with centrally located was even higher in limb muscles of 4-month-old *dy^3K^*/δE3 animals, indicating that pathology worsens over time (Fig. 6A and data not shown). Nevertheless, these results indicate that *dy^3K^*/δE3 muscles undergo damage but that the constant regeneration and muscle mass is maintained with age. Moreover, the diaphragm did not undergo degeneration/regeneration cycles and its morphology appeared near normal in 5-week-old and 4-month-old animals (Fig. 6A–C). *Dy^3K^/dy^3K^* diaphragm at 4–6-weeks of age displayed about 16% of regenerated muscle fibers with central nuclei. A significant reduction was found in *dy^3K^*/δE3 diaphragm, both in young and older animals and the numbers did not differ significantly from wild-type diaphragm (Fig. 6B). We also determined the muscle fiber size in 4–6-week-old diaphragm muscle. The fiber size distribution was shifted towards smaller fiber sizes in *dy^3K^/dy^3K^* animals, compared with wild-type muscles. Notably, the shift was largely prevented in *dy^3K^*/δE3 muscles (Fig. 6C).

**Figure 7:**
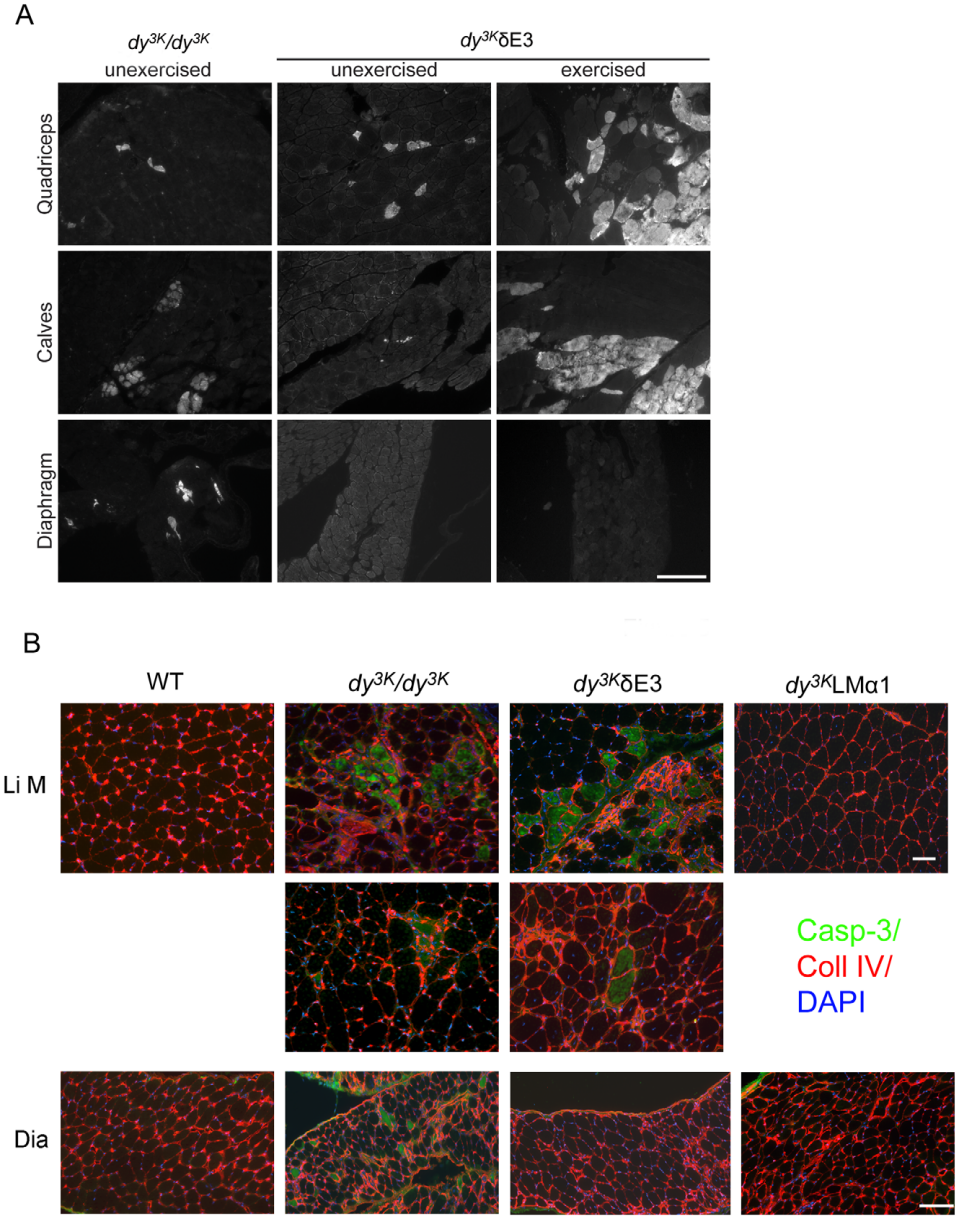
EBD staining of unexercised and exercised muscles and caspase-3 immunostaining. (A) Three- to 5-week-old *dy^3K^*/*dy^3K^* mice (not exercised) display a few EBD-positive fibers. Also, unexercised *dy^3K^*/δE3 limb muscles display few fibers positive for EBD, whereas hardly any are detected in *dy^3K^*/δE3 diaphragm. *Dy^3K^*/*dy^3K^* mice were not in the condition to be exercised on the treadmill, but 7–13-week-old *dy^3K^*/δE3 mice were analyzed for EBD uptake upon exercise. Increased uptake of EBD is seen in exercised *dy^3K^*/δE3 limb muscles, but truncated LMα1 chain prevents exercise-induced injury in diaphragm. Bar, 200 µm. (B) Robust expression of caspase-3 (green) in the fibers from *dy^3K^/dy^3K^* and *dy^3K^*/δE3 limb muscles indicated ongoing apoptosis in a large group of fibers (top Li M panel), or in single fibers (lower Li M panel). Overexpression of full-length LMα1 chain prevented the cell death in LMα2 chain deficient limb muscles. In contrast to limb muscles, only *dy^3K^/dy^3K^* diaphragm (Dia) contained apoptotic fibers, whereas the overexpression of both δE3LMα1 and full-length LMα1 chain prevented apoptosis in LMα2 chain deficient diaphragms. DAPI (blue) and an antibody against collagen IV (red) were used to co-visualize apoptotic fibers. Four animals from each group were analyzed. Bars, 50 µm.

To demonstrate functional benefit conferred by the truncated LMα1 chain in diaphragm, we subjected *dy^3K^*/δE3 mice to downhill treadmill exercise and sarcolemmal integrity was evaluated by Evans blue dye (EBD) accumulation. It has previously been shown that only occasional EBD-positive fibers are found in *dy/dy* muscles [40]. In agreement with these results, we also detected a few EBD-positive fibers in unexercised *dy^3K^/dy^3K^* muscles. We also observed a few EBD-positive fibers in unexercised *dy^3K^*/δE3 limb muscles, but almost none in *dy^3K^*/δE3 diaphragm (Fig. 7A). While it was not possible to exercise *dy^3K^/dy^3K^* animals, *dy^3K^*/δE3 limb muscles were susceptible to exercise-induced sarcolemmal injury as evidenced by increased uptake of EBD. Interestingly, downhill running induced very little damage in *dy^3K^*/δE3 diaphragm (Fig. 7A). Although EBD uptake in exercised *dy^3K^*/δE3 limb muscles varied, both between animals and opposing limbs within the same animal, the diaphragm was consistently unaffected. Hence, truncated LMα1 chain prevents exercise-induced injury in diaphragm but not in limb muscles, indicating that different muscles have different requirements for LMα1LG4-5 domains.

**Figure 8:**
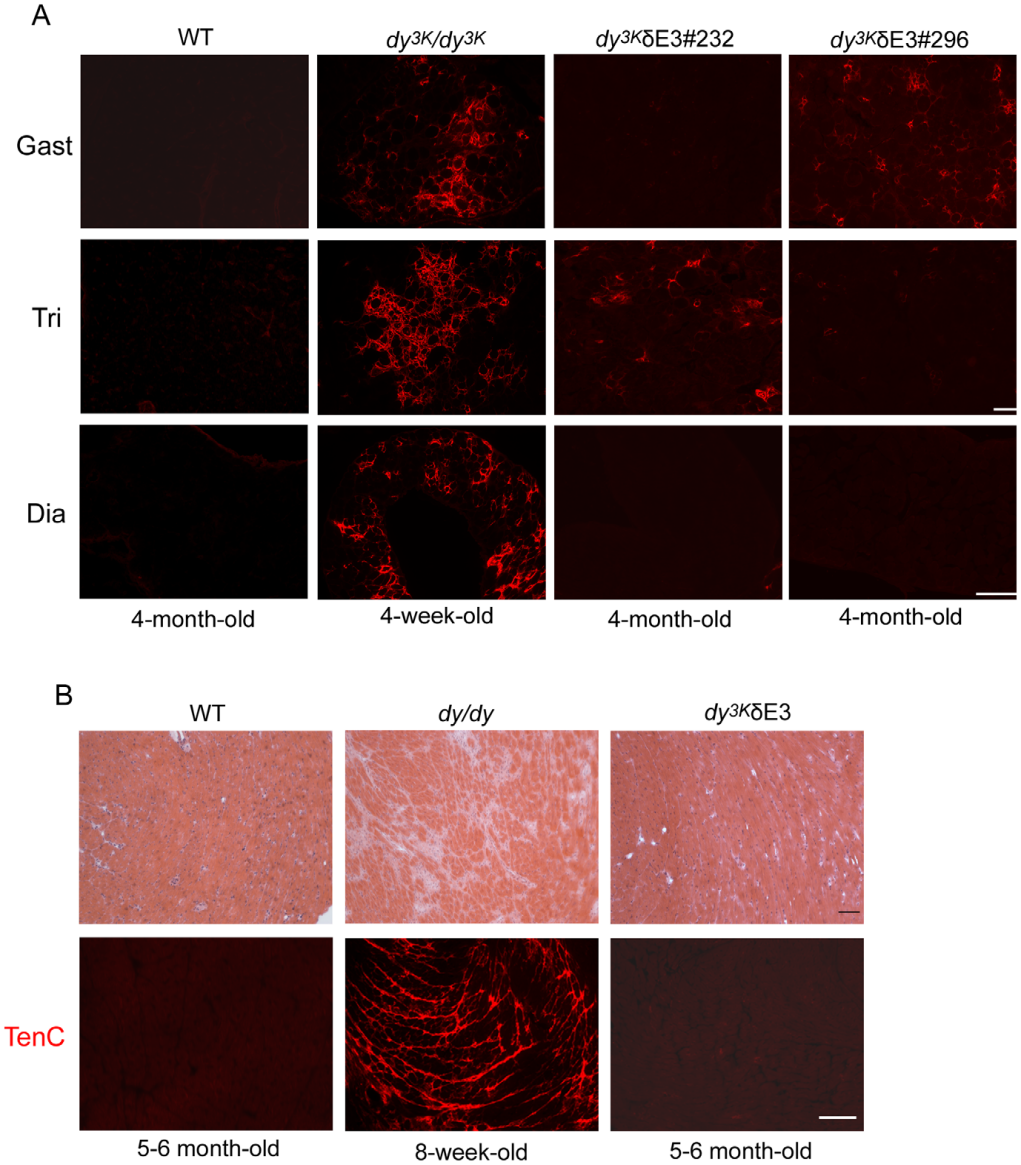
Analyses of fibrosis in skeletal muscle and heart. (A) Different wild-type (4-month-old), *dy^3K^/dy^3K^* (4-week-old) and *dy^3K^*/δE3 (4-month-old) muscles (gastrocnemius, triceps, diaphragm) were stained with an antibody against tenascin-C. Occasionally tenascin-C is present in interstitial matrix of limb *dy^3K^*/δE3 muscles, but it is absent from diaphragm. Note extensive tissue fibrosis in *dy^3K^/dy^3K^* muscles. Four *dy^3K^*/δE3 animals were analyzed. Bars, 50 µm. (B) Hematoxylin and eosin staining (upper panel) of hearts from wild-type (5–6-month-old), *dy/dy* (8-week-old) and *dy^3K^*/δE3 (5–6-month-old) mice. Hearts from *dy/dy* mice displayed localized or extensive fibrosis in the ventricular wall. *Dy^3K^*/δE3 hearts did not exhibit any defects and looked as wild-type controls. Tenascin-C immunolabelling confirms the presence of fibrotic lesions in *dy/dy* hearts and their absence in *dy^3K^*/δE3 hearts (lower panel). Three animals from each group were analyzed. Bars, 50 µm.

The phenomenon of progressive muscle fiber damage in the limbs was further underscored by caspase-3 staining. Apoptosis has been shown to contribute to the severe dystrophic changes in muscles from MDC1A patients and LMα2 chain deficient mice [2], [41], [42]. In both *dy^3K^/dy^3K^* and *dy^3K^*/δE3 muscles either single caspase-3 positive apoptotic fibers were detected or apoptosis was more robust (Fig. 7B). In contrast, the muscles from LMα2 chain deficient mice overexpressing full-length LMα1 chain (*dy^3K^*LMα1) were free of apoptotic fibers (no caspase-3 staining was observed, Fig. 7B). Interestingly, apoptosis did not take place in *dy^3K^*/δE3 diaphragms, whereas apoptotic fibers were present in diaphragms from *dy^3K^/dy^3K^* mice (Fig. 7B). This data strongly suggests that LMα1LG4-5 protects limb muscles from apoptosis, most probably via dystroglycan binding, whereas truncated LMα1 chain is sufficient to prevent apoptosis in diaphragm muscle fibers.

Regardless of apoptotic cell death, muscle replacement with connective tissue, so evident in *dy^3K^/dy^3K^* mice [25], was not very obvious in *dy^3K^*/δE3 muscles (Fig. 6A). This tendency was also demonstrated by tenascin-C staining. Tenascin-C has been shown to be upregulated and extends to the interstitium between muscle fibers in *dy/dy* and *dy^3K^/dy^3K^* mice [25], [43]. Some muscles from different *dy^3K^*/δE3 animals showed moderate upregulation of tenascin-C (Fig. 8A, two individuals are shown, four animals were analyzed). However, tenascin-C expression was less pronounced than in *dy^3K^/dy^3K^* muscles. Also, some *dy^3K^*/δE3 limb muscles did not display tenascin-C upregulation (Fig. 8A). Moreover, diaphragm did not show any signs of fibrosis (Fig. 8A).

Cardiomyopathy is not a major feature of MDC1A [1]. However, 2-month-old *dy^W^/dy^W^* hearts show infiltration of connective tissue [44]. *Dy^3K^/dy^3K^* mice probably die too early in order to develop heart fibrosis (data not shown). Therefore, we compared 5–6-month-old *dy^3K^*/δE3 hearts with hearts from 8-week-old *dy/dy* mice, which show massive fibrosis in the ventricle wall (Fig. 8B). As demonstrated by hematoxylin and eosin staining, *dy^3K^*/δE3 hearts did not display any fibrotic lesions (Fig. 8). This trend was further confirmed by absence of tenascin-C staining (Fig. 8B).

In summary, LMα1LG4-5 domains are important for securing the mechanical stability of limb muscle fibers in LMα2 chain deficiency, most probably by binding to dystroglycan. Interestingly, LMα1LG4-5 domains are not involved in improvement of diaphragm and heart muscle morphology, indicating that other sites of LMα1 chain (most likely integrin α7β1 binding modules) are responsible for functional replacement of LMα2 chain in these muscles.

### Skeletal muscle regeneration is not impaired in *dy^3K^*/δE3 mice

**Figure 9:**
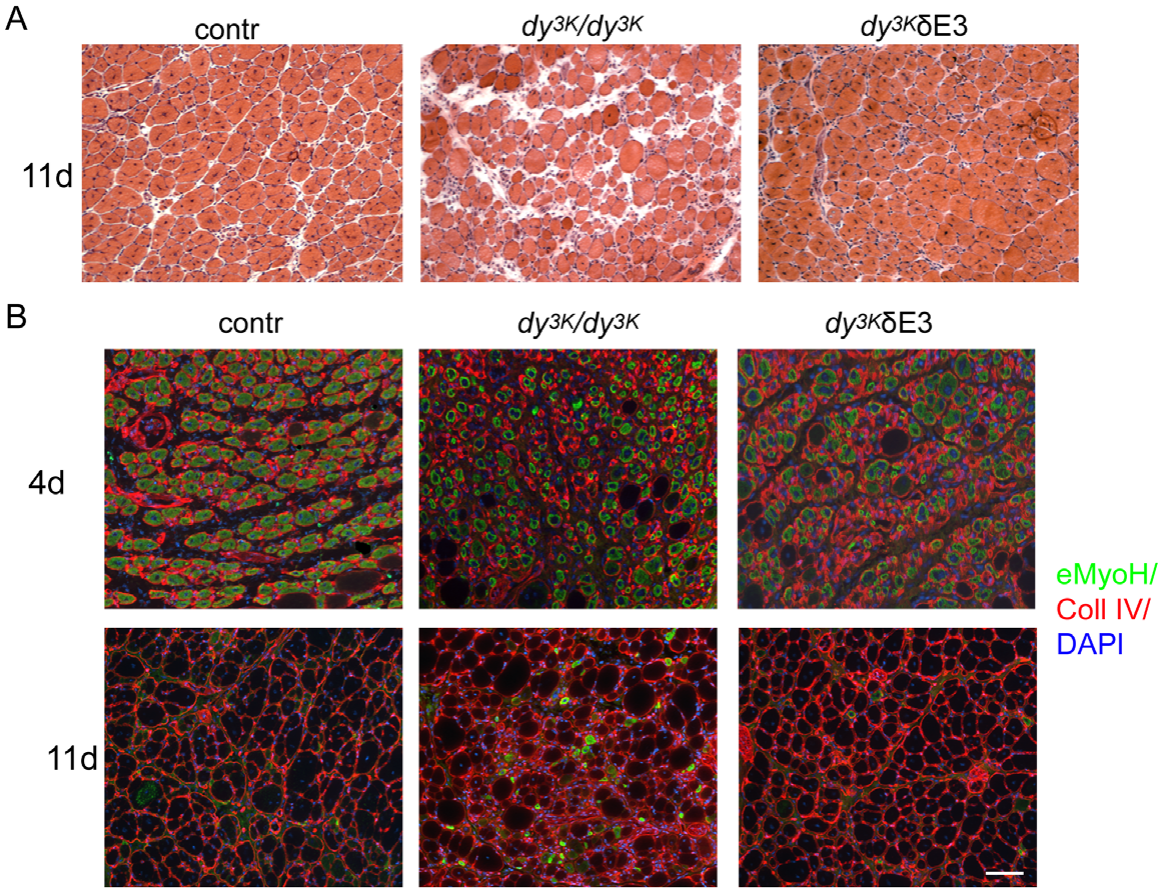
Analyses of skeletal muscle regenerative properties subjected to cardiotoxin injection. (A) Hematoxylin and eosin staining of tibialis anterior from control (2–3-month-old), *dy^3K^/dy^3K^* (3-week-old) and *dy^3K^*/δE3 (2–3-month-old) 11 days post cardiotoxin injection. Regenerating *dy^3K^*/δE3 muscles morphologically look like regenerating control muscles, whereas regeneration in *dy^3K^/dy^3K^* mice is delayed. (B) Immunostaining revealing the presence of embryonic myosin heavy chain (eMyoH) as the sign of active regeneration (green). Collagen IV (Coll IV) antibody (red) and DAPI nuclear marker (blue) were chosen to co-visualize regenerating fibers. Four-days post injection (upper panel) all analyzed muscles express embryonic myosin. Fibers from *dy^3K^/dy^3K^* mice are smaller. Eleven-days post injection (lower panel) control and *dy^3K^*/δE3 tibialis anterior do not express embryonic myosin. Embryonic myosin is occasionally present in some *dy^3K^/dy^3K^* fibers. *Dy^3K^/dy^3K^* tibialis anterior does not show regular morphology and displays dystrophic, disorganized pattern with small and big muscle fibers. Three animals from each group were analyzed. Bars, 50 µm.

Since muscle regeneration seemed to be continuously maintained in *dy^3K^*/δE3 limb muscles (Fig. 6A), we next analyzed their regenerative properties in more detail. We injected 2–3-month-old control, *dy^3K^*/δE3 mice and 3-week-old *dy^3K^/dy^3K^* tibialis anterior with cardiotoxin to induce muscle damage and trigger muscle regeneration. Four days after injection many new fibers had reformed in all mice examined (data not shown). These fibers expressed embryonic myosin heavy chain, indicating an ongoing regeneration (Fig. 9B). Surprisingly, the regeneration process clearly took place in the absence of LMα2 chain (although newly formed muscle cells in *dy^3K^/dy^3K^* tibialis anterior were rather small) (Fig. 9B). Tibialis anterior from *dy^3K^*/δE3 mice also showed normal initial regeneration, comparable to control mice. Most importantly, after 11 days post injection, *dy^3K^*/δE3 muscles displayed the regeneration pattern characteristic for control mice and they were not distinguishable from each other (Fig. 9A). Injected *dy^3K^*/δE3 tibialis anterior muscles were tightly packed with big fibers. Also, the expression of embryonic myosin heavy chain was not detected after 11 days (Fig. 9B). This data confirms that regeneration in the presence of truncated LMα1 chain is characterized with high capacity and maintenance. The regeneration in *dy^3K^/dy^3K^* mice was delayed and not as well-organized as in control and *dy^3K^*/δE3 animals, since the muscle fibers in LMα2 chain deficient mice appeared to be less packed and surrounded by connective tissue (Fig. 9A). Also, single fibers still expressed embryonic myosin heavy chain.

In summary, these data provide more insight into mechanism of muscle regeneration in LMα2 chain deficiency and indicate that LMα1 chain deprived of LG4-5 domains ensures proper regeneration. Therefore, binding to dystroglycan is not essential to ensure sufficient muscle regeneration and its maintenance.

### LMα1LG4-5 is essential for myelination in peripheral nervous system in LMα2 chain deficiency

**Figure 10:**
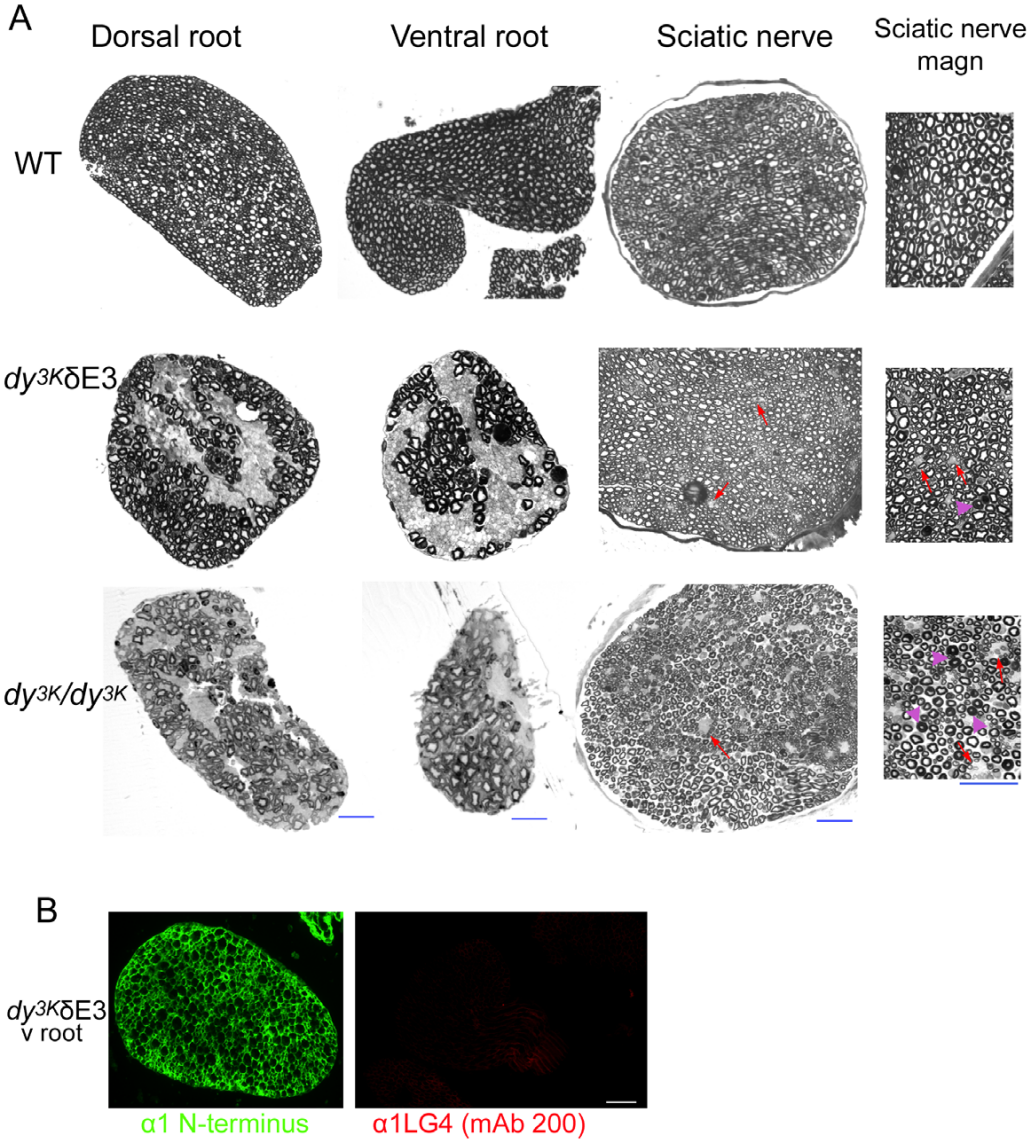
Analyses of myelination in peripheral nervous system. (A) Toluidine blue staining of ventral and dorsal roots and sciatic nerves from 2–4-month-old normal and *dy^3K^*/δE3 mice and 5-week-old *dy^3K^/dy^3K^* animals. Myelination defects are clearly visible in *dy^3K^*/δE3 and *dy^3K^/dy^3K^* spinal roots with distinct and wide-spread unmyelinated axons bundles. Occasional unmyelinated axon bundles are also detected in sciatic nerve of *dy^3K^*/δE3 and *dy^3K^/dy^3K^* mice (indicated with arrows). Arrowheads denote tomacula. (B) Truncated LMα1 chain is present in *dy^3K^*/δE3 spinal roots as demonstrated by immunostaining using the antibody against N-terminal (green) and LG4 domain no staining). Four animals from each group were analyzed. Bars, 25 µm.

MDC1A patients as well as *dy^3K^/dy^3K^* mice display dysmyelination neuropathy that leads to reduced conduction velocity of nerve impulses [45]–[47]. Unmyelinated axon bundles are prominent especially in spinal roots of LMα2 chain deficient mice. We have demonstrated before that overexpression of full-length LMα1 chain in *dy^3K^/dy^3K^* peripheral nervous system largely corrects myelination defects [27]. *Dy^3K^*/δE3 mice display hindleg paralysis and motor dysfunction. Morphology analyses of spinal roots and sciatic nerves confirmed that overexpression of truncated LMα1 chain did not correct the phenotype of the proximal part of peripheral nervous system. In spite of the presence of truncated LMα1 chain in both dorsal and ventral roots, large areas with unmyelinated axons (indicating incomplete axonal sorting) were evident in *dy^3K^*/δE3 mice (Fig. 10). Similar bundles of naked, unmyelinated axons have also been described in dorsal and ventral roots of *dy^3K^/dy^3K^* mice [27]. Importantly, this process was fully prevented upon overexpression of full-length LMα1 chain in LMα2 chain deficient peripheral nervous system [27], suggesting a role for LG4-5 domains in myelination processes.

**Figure 11:**
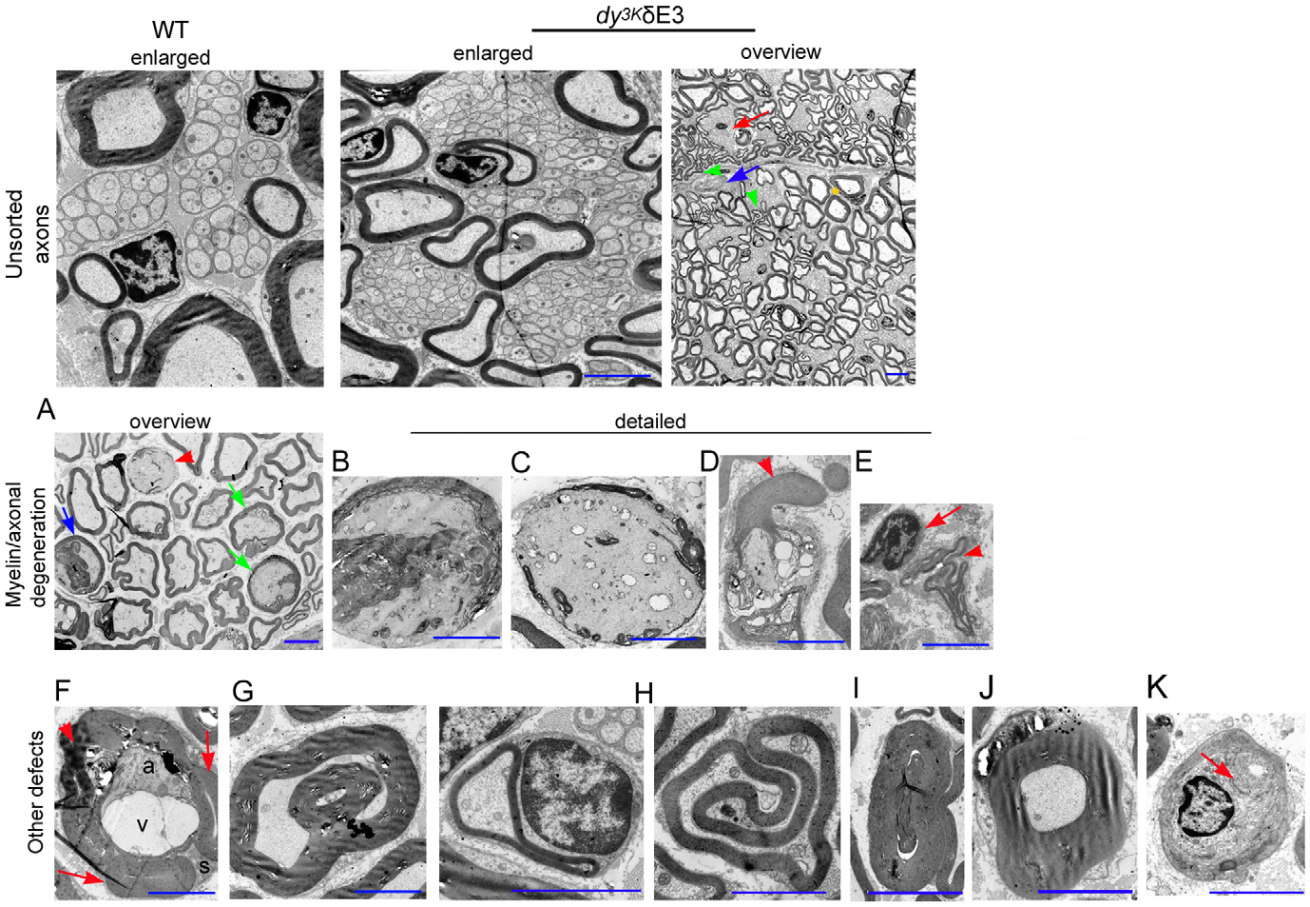
Detailed analyses of morphology and properties of 2–4-month-old *dy^3K^*/δE3 sciatic nerves (electron microscopy). Top panel: Unsorted axons in wild-type (WT) and *dy^3K^*/δE3 sciatic nerves. Most of bundles of unmyelinated axons are bigger in *dy^3K^*/δE3 mice (enlarged panels). Apart from unsorted axons (red arrow, overview panel), many compressed, ovoid axons, often with convoluted outfoldings and redundant loops are seen (green arrowhead). Yet, numerous normally shaped and myelinated axons are present (yellow star). Single macrophages were detected (blue arrow). Middle panel: Myelin defects linked to axonal degeneration. (A) Overview of a pathological area with different stages of myelin abnormalities, myelin degradation and axonal degeneration. Red arrowhead - degenerating axon. Blue arrow - degraded interaxonal myelin leading to axon degeneration. Green arrow - axons with vesicular or lamellar myelin debris (intrusions) and dense bodies, often being signs of early stage of degeneration. (B–E) Detailed photos of different forms of degenerating axons found in various areas of sciatic nerve. (B) Degenerating axon with interaxonal myelin debris. (C) An almost completely demyelinated nerve fiber is filled with dilated smooth endoplasmic reticulum and degenerated mitochondria and undergoes degeneration. (D) Granular myelin degeneration with numerous myelin breaks. Arrowhead indicates myelin outfoldings/redundant loop formation. (E) Axonal degeneration forgoes myelin degradation as indicated by loose non-degraded myelin swirls. Schwann cell detached from empty myelin is indicated with arrow. Bottom panels: various axonal and myelin distortions rooting from incorrect myelination process and/or disruption of Schwann cell properties after myelination. (F) One Schwann cell (S) contains thinly myelinated axon (a) with vacuole (v), swollen myelin debris (arrowhead) and thickened myelin sheaths of minute axons (arrow) or myelin outfoldings. (G) Satellite myelinated axon within a bigger axon or excessive intramyelin fold. Myelin outfoldings and satellite myelination seen in F and G may result from impaired myelination process. (H) Redundant loop formation. (I) Hypermyelination due to excessive redundant loop formation. (J) Tomacula. (K) Onion bulb. Arrow indicates an almost demyelinated axon. Bar, 3 µm.

Although myelination took place in the distal part of *dy^3K^*/δE3 peripheral nervous system, sciatic nerve morphology was only partially rescued compared to *dy^3K^*/*dy^3K^* mice. Bundles of unsorted unmyelinated axons have been reported in *dy^3K^*/*dy^3K^* sciatic nerve [27] (see Fig. 10). Smaller, yet clearly visible patches of unsorted axons were also detected in *dy^3K^*/δE3 sciatic nerves (Fig. 10 and 11). While occasional unmyelinated axons are present in normal animals (Fig. 11, top panel) and they are known to be part of a healthy nerve, the bundles present in *dy^3K^*/δE3 nerves were clearly bigger (Fig. 11, top panel) and more frequent (data not shown), than in control mice. Tomacula (thickened myelin sheaths) was observed in *dy/dy* mice [48] and we also detected these hypermyelinated axons in *dy^3K^*/*dy^3K^* animals (Fig. 10). Fewer tomacula were seen in *dy^3K^*/δE3 mice (Fig. 10). Electron microscopy analyses of 2–4-month-old *dy^3K^*/δE3 sciatic nerves revealed a whole spectrum of pathologies. Apart from axons with normal appearance (Fig. 11, top panel, yellow star), many axons with myelin distortion and/or abnormal ovoid shape were detected, especially in the animals affected more severely with paralysis (Fig. 11, top panel, 3^rd^ overview photo; middle panel and bottom panel). The post-myelination pathologies leading to axonal degeneration (Fig. 11A–E) included: myelin degradation, axon demyelination (B,C), myelin intrusions (A), excessive myelin outfoldings (A,D) and redundant loops (H). Degenerated axons often resembled Wallerian degeneration (Fig. 11E) [49]. Many Schwann cells detached from degenerating axons (Fig. 11E, arrow) and showed anomalous, most probably pre-apoptotic phenotype. Further abnormalities included presence of intra-axonal vacuoles (Fig. 11F), myelin infoldings (Fig. 11G), different forms of hypermyelination (Fig. 11I and J) and occasional onion bulbs (several concentric layers of Schwann cell cytoplasm around an axon, leading to demyelination) (Fig. 11K). Schwann cells myelinating more than one axon (satellite axons) were found (Fig. 11F and G). This may point towards defective behavior of Schwann cells and as a consequence a defective myelination process. Many of the described abnormalities have not been associated with LMα2 chain deficiency before. However, redundant loop formation is characteristic for *dy/dy* mice [48], and we also found many axons with redundant loops (Fig. 11H, and top panel overview). Redundant loop formation by Schwann cells and collapsing myelin that form ovoid, flat axons could contribute to axonal necrosis [50]. In conclusion, it is possible that upon LMα2 chain deficiency and in the absence of full-length LMα1 chain, Schwann cells acquire pathological properties and perform abnormal myelination. Furthermore, with age these Schwann cells could affect correctly assembled myelin layers, subsequently leading to axonal neuropathy.

These data show that the presence of truncated LMα1 chain did not prevent the possible age-related progression of pathological processes in *dy^3K^/dy^3K^* distal peripheral nervous system. Therefore, LMα1LG4-5 has a crucial role not only for myelination of the spinal roots, but also for correct myelination, maintenance of myelin, proper axon-Schwann cell interaction and peripheral nerve homeostasis in the distal peripheral nervous system. Various myelin and Schwann cell abnormalities have been shown to contribute to demyelination in different neuropathies [51]. Likewise, the myelin defects described above could influence the severity of observed neuropathy.

### Basement membranes are not fully restored in the presence of truncated LM α1 chain

**Figure 12:**
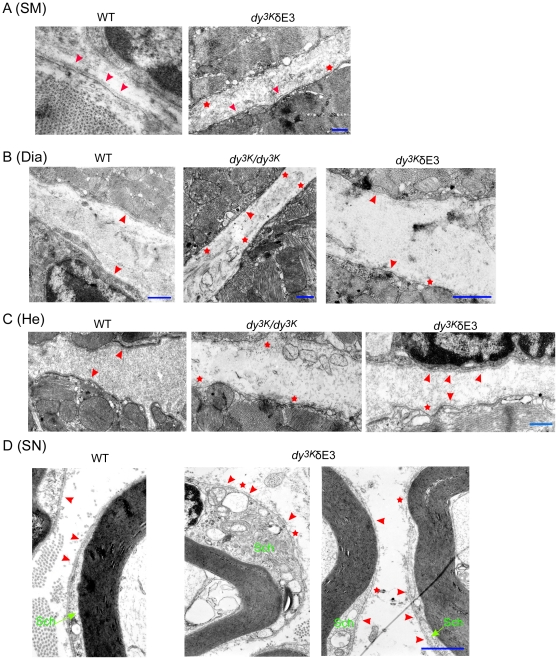
Basement membranes in the neuromuscular system in the absence of LMα1LG4-5. Electron microscopy of (A) limb skeletal muscle (wild-type and *dy^3K^*/δE3); (B) diaphragm (wild-type, *dy^3K^/dy^3K^* and *dy^3K^*/δE3); (C) heart (wild-type, *dy^3K^/dy^3K^* and *dy^3K^*/δE3); (D) sciatic nerve (wild-type and *dy^3K^*/δE3). In *dy^3K^*/δE3 limb skeletal muscle basement membranes had patchy appearance as compared to continuous basement membranes in wild-type samples (A) (arrowheads, in all figures). Stars depict the areas with lack of basement membrane in all figures. In *dy^3K^/dy^3K^* diaphragm basement membranes are either patchy or completely absent. Presence of truncated LMα1 chain partially restores basement membranes in the diaphragm (B). Similarly, in LMα2 chain deficient heart basement membranes are disrupted and partially restored upon δE3LMα1 chain overexpression (C). Basement membranes were locally patchy around *dy^3K^*/δE3 Schwann cells (SCh), but also sometimes continuous throughout longer distances (D). Four animals from each group were analyzed. Bars, 400 nm.

LMα2 chain deficiency results in disrupted basement membranes around muscle and Schwann cells [2], [25], [27], [30], [46], [52], [53]. Overexpression of full-length LMα1 chain largely restores basement membranes in the neuromuscular system of *dy^3K^/dy^3K^* mice [25], [27]. In *dy^3K^*/δE3 mice, basement membrane assembly was only partially re-established. Both in sciatic nerves and especially in skeletal muscle, basement membranes had a patchy appearance (Fig. 12, A and D). In diaphragm muscle and heart, despite significant morphological improvement, basement membranes were also locally discontinuous (although to a lesser extent than in limb muscle), suggesting that the improvement of the phenotype is not entirely related to intact basement membranes in these organs. Nevertheless, basement membranes in *dy^3K^/dy^3K^* diaphragm and heart were more disrupted than in *dy^3K^*/δE3 animals (Fig. 12, B and C).

In summary, these data show that LMα1LG4-5 is partially required for basement membrane assembly and cell surface anchorage in the neuromuscular system.

## Discussion

In this paper, we investigated the roles of LM C-terminal globular domains (and accordingly LM receptors dystroglycan and integrin) in muscle and nerve and analyzed the molecular mechanisms underlying LMα1 chain mediated rescue of LMα2 chain deficiency.

### LMα1LG4-5 is dispensable for improvement of diaphragm and heart muscles, but not limb muscles in LMα1 chain rescued mice

Overexpression of LMα1 chain lacking LG4-5 domains in *dy^3K^/dy^3K^* mice resulted in significantly prolonged lifespan (at least tripled compared with *dy^3K^/dy^3K^* mice). Cardiopulmonary complications are often responsible for the early death in dystrophic patients but cardiomyopathy is not a common feature of LMα2 chain deficiency [1]. Considering that a severely dystrophic diaphragm will lead to pulmonary dysfunction, it is quite likely that the improved diaphragm in *dy^3K^*/δE3 mice accounts for the increased survival, although we can not completely exclude that the expression of truncated LMα1 in other tissues (e.g. heart) is beneficial. Importantly, integrin α7B subunit is absent from *dy^3K^/dy^3K^* sarcolemma, but reconstituted in *dy^3K^*/δE3 muscles. Hence, we propose that prolonged lifespan is secured via LMα1LG1-3 binding, most probably to integrin α7β1, in the diaphragm and possibly in the heart.

Interestingly, while LMα1LG4-5 turned out to be dispensable for diaphragm and heart muscle, overexpression of LMα1 chain devoid of LG4-5 did not secure the complete correction of LMα2 chain deficient limb muscles. Although it is not surprising that LMα2 chain deficient peripheral nerve and muscle could respond differently to δE3LMα1 overexpression, it is somewhat unexpected that limb muscles and diaphragm would not be spared to the same degree, indicating an important difference in their properties or molecular signature in response to lack of a single protein domain. Our results also point toward diverse roles of LMα1LG4-5 when expressed in different muscle groups. For example, apoptosis has been shown to contribute to LMα2 chain deficient pathogenesis [54], [55]. In limb skeletal muscle, LMα1LG4-5 appeared to be critical for prevention of apoptosis of muscle fibers. However, this was not the case in diaphragm. Integrin α7β1 has been considered to be the major mediator of myofiber survival [29]. Now, we suggest that also LM binding to dystroglycan prevents apoptosis in limb muscle fibers. In support of this notion, dystroglycan binding to LMα2 chain has been shown to protect muscle cells in culture from apoptosis [56]. Yet, in some muscles, (e.g. diaphragm) integrin α7β1 could be the key player in apoptosis prevention.

### LMα1LG4-5 is not involved in muscle regeneration in LMα1 chain rescued mice

Skeletal muscle regeneration depends on satellite cells, which express both dystroglycan and integrin α7β1 [10], [57]. In spite of muscle damage and cell death, dy^3K^/δE3 muscles were able to regenerate and maintain muscle mass, both in normal conditions and when subjected to cardiotoxin injection. Also, mini-agrin increases the regenerative capacity of LMα2 chain deficient muscles. Since mini-agrin binds dystroglycan (rather that integrin α7β1), it is hypothesized that mini-agrin binding to dystroglycan is responsible for the restored regeneration [58], [59] and it has been demonstrated that dystroglycan activity in satellite cells is crucial for the maintenance of regeneration [10]. Yet, integrin α7 chain is also involved in skeletal muscle regeneration, as α7 integrin-null mice subjected to cardiotoxin injections exhibit a profound delay in muscle regeneration [57]. Hence, integrin α7 chain is most likely responsible for efficient muscle regeneration in dy^3K^/δE3 mice since the dystroglycan binding domain is missing. We propose that the most aggravating step in MDC1A might be the lack of efficient regeneration due to abolished LMα2-integrin α7 interaction rather than impaired LMα2-dystroglycan interaction.

### LMα1LG4-5 is vital for myelination in peripheral nerve in LMα1 chain rescued mice

None of the neuronal symptoms that occur in LMα2 chain deficiency were ameliorated by δE3LMα1 overexpression. This data together with our previous work [27] indicates a very important role for LMα1LG4-5 in LMα1 chain rescued peripheral nervous system. Interestingly, the phenotype of *dy^3K^/dy^3K^* and *dy^3K^*/δE3 peripheral nervous system does not resemble the phenotype of any conditional knockout mice, where major LM receptors (dystroglycan, integrins β1 and β4) were depleted from Schwann cells [18]–[20], [60]. Furthermore, genetic inactivation of the α7 integrin chain does not affect peripheral nerve morphology and function [60]. Therefore, those receptors might just regulate the LMα2 chain/LMα1 chain interaction together with other receptors. Heparan sulfate proteoglycans syndecans presumably bind LMα1 via the LG4 domain [61] and are enriched in Schwann cells [62], but syndecan-null mice do not display peripheral nerve defects [63]. Also, sulfatides have been shown to bind LMα1LG4-5 [64] and LMα2LG4-5 [65], [66] and to be expressed in peripheral nerves [67], where they mediate basement membrane assembly and dystroglycan and integrin signaling [68]. Strikingly, lack of sulfatides and galactocerebrosides (another type of glycolipids) in mice results in similar myelin abnormalities in central nervous system as in *dy^3K^*/δE3 distal peripheral nervous system. Hence, the LM receptor might belong to glycolipids [69]–[71]. Furthermore, monosialoganglioside GM1 has been shown to bind LM-111 and promote neurite outgrowth [72]. Therefore, the identification of a peripheral nerve LM receptor is an exciting task.

### Basement membrane assembly in LMα1 chain rescued mice requires LMα1LG4-5

In early studies of LMα2 chain deficiency, lack of basement membranes was considered to be deleterious to the muscle fibers [2], [52], [73], [74] and to represent one of the MDC1A pathogenic mechanisms. Consequently, the approach of basement membrane restoration has been hypothesized to be beneficial for the improvement of the dystrophic muscle phenotype [25], [28], [44], [53]. Yet, continuous basement membranes are not strictly required for myelination in peripheral nervous system [46], [75]. Likewise, basement membranes are also patchy or less dense in *dy^3K^*/δE3 mice diaphragm and heart muscle, indicating that continuous basement membranes are not vital for the complete correction of the dystrophic phenotype.

Our data helps to further understand the involvement of LMα1LG4-5 and LG1-3 in basement membrane assembly and point toward interesting basement membrane scaffolding mechanisms in the neuromuscular system in the absence of LMα1LG4-5. Exogenous LMα1LG4-5 has been shown to totally abolish the formation of basement membranes *in vitro* where it selectively blocked the cell-surface accumulation of a LM network [68], . In our *in vivo* model, despite lack of LMα1LG4-5, basement membranes showed only partial defects in cell surface anchoring. It is not excluded that integrins or other receptors that bind LMα1LG1-3, partially could compensate for lack of LMα1LG4-5 domain and dystroglycan/sulfatide binding and anchor the LM network to the cell surface. This accumulation, however, is not as efficient as in the presence of full-length LMα1 chain or mini-agrin [25], [27], [44], [53], as basement membranes appear to be continuous only locally in *dy^3K^*/δE3 mice. Therefore, it is possible that all LMα1LG domains and the cooperation between different LMα1LG1-5 receptors are important for the assembly of continuous basement membranes *in vivo*. This hypothesis is further substantiated in McKee et al., where all LG domains were shown to support LM tethering to cell surface [78], [79]. However, very recent data by Han *et al*., [15] confirms that dystroglycan, but not integrin α7β1, is involved in basement membrane anchorage and maintenance (rather than actual assembly) in muscle. Therefore, LMα1LG4-5 binding to dystroglycan could be important not only for basement membrane assembly in the muscle, but also for its maintenance.

## Supporting Information

Figure S1 Expression of δE3LMα1 chain in limb skeletal muscle (SM), peripheral nerve (SN) and heart (He) of δE3 transgenic mice from lines No. 3 and 4. The two antibodies to detect truncated LMα1 chain were mAb200 and 1057+, which bind LG4 and N-terminal domains, respectively. Mosaic expression of δE3LMα1 chain was detected in transgenic neuromuscular tissues. Wild-type (WT) mice and full-length LMα1 chain transgenic animals (LMα1TG) were used as controls. Bars, 50 µm.
(3.82 MB TIF)

Figure S2 Immunostaining of LMα4 and α5 chains. Cross-sections of quadriceps femoris (Quad), triceps brachii (Tri) and diaphragm (Dia) from 6-week-old wild-type, dy3K/dy3K and dy3K/δE3 mice were stained with antibodies against LMα4 chain (A) and α5 chain (B), respectively. Expression of LMα4 and α5 chains is increased at the muscle basement area in dy3K/dy3K mice and remains increased in dy3K/δE3 muscles. Four dy3K/δE3 animals were analyzed. Bar, 50 µm.
(3.67 MB TIF)

Figure S3 The numbers of fibers in a randomly selected area is not significantly different between the genotypes.
(0.20 MB TIF)
