# Supplementary figures and images for: Distinct Roles for Laminin Globular Domains in Laminin α1 Chain Mediated Rescue of Murine Laminin α2 Chain Deficiency
Source: PLoS One. 2010 Jul 19;5(7):e11549. doi: 10.1371/journal.pone.0011549 (PMC2906511; doi:10.1371/journal.pone.0011549)

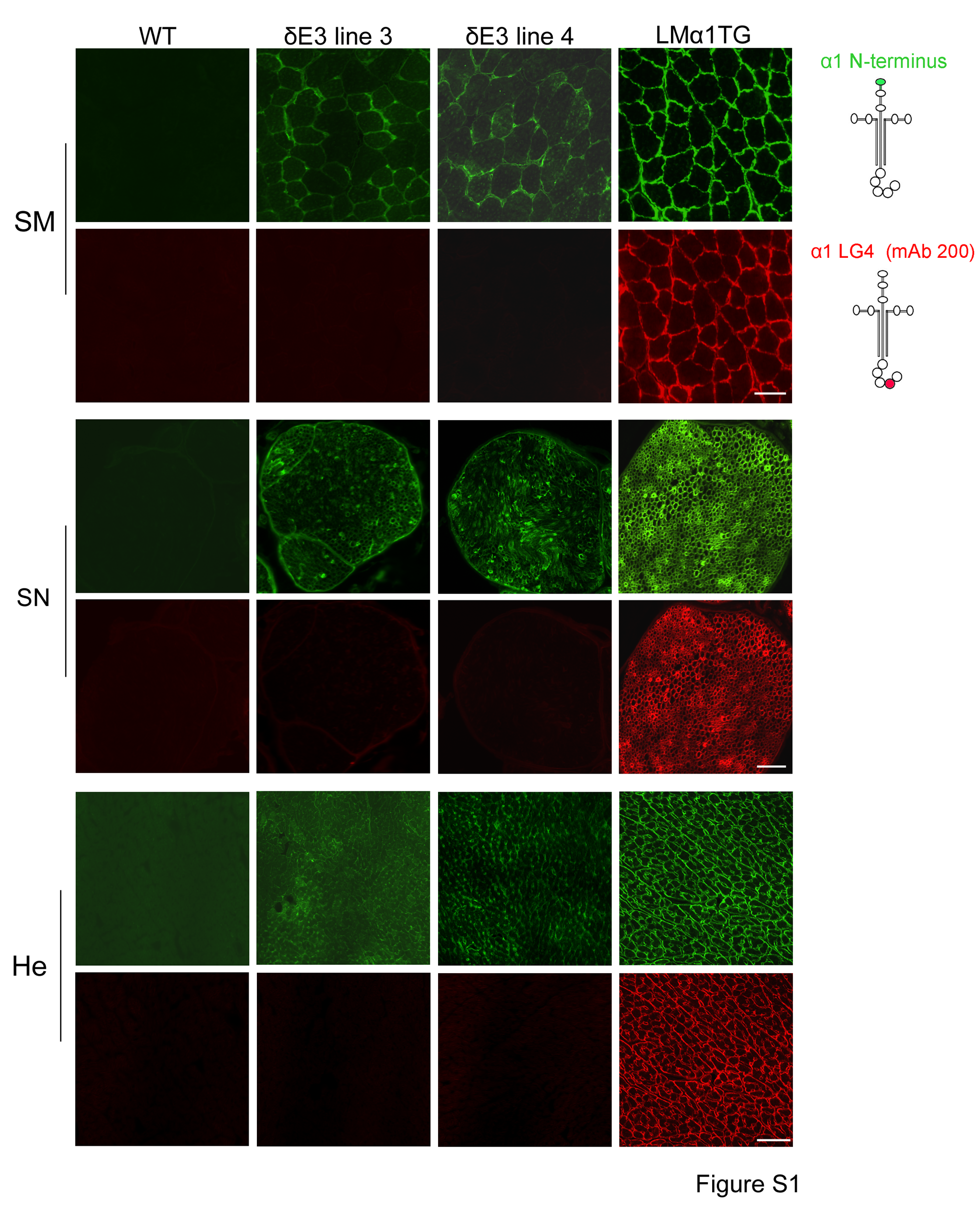

Supplement: Figure S1 — Expression of δE3LMα1 chain in limb skeletal muscle (SM), peripheral nerve (SN) and heart (He) of δE3 transgenic mice from lines No. 3 and 4. The two antibodies to detect truncated LMα1 chain were mAb200 and 1057+, which bind LG4 and N-terminal domains, respectively. Mosaic expression of δE3LMα1 chain was detected in transgenic neuromuscular tissues. Wild-type (WT) mice and full-length LMα1 chain transgenic animals (LMα1TG) were used as controls. Bars, 50 µm. (3.82 MB TIF) [file pone.0011549.s001.tif]

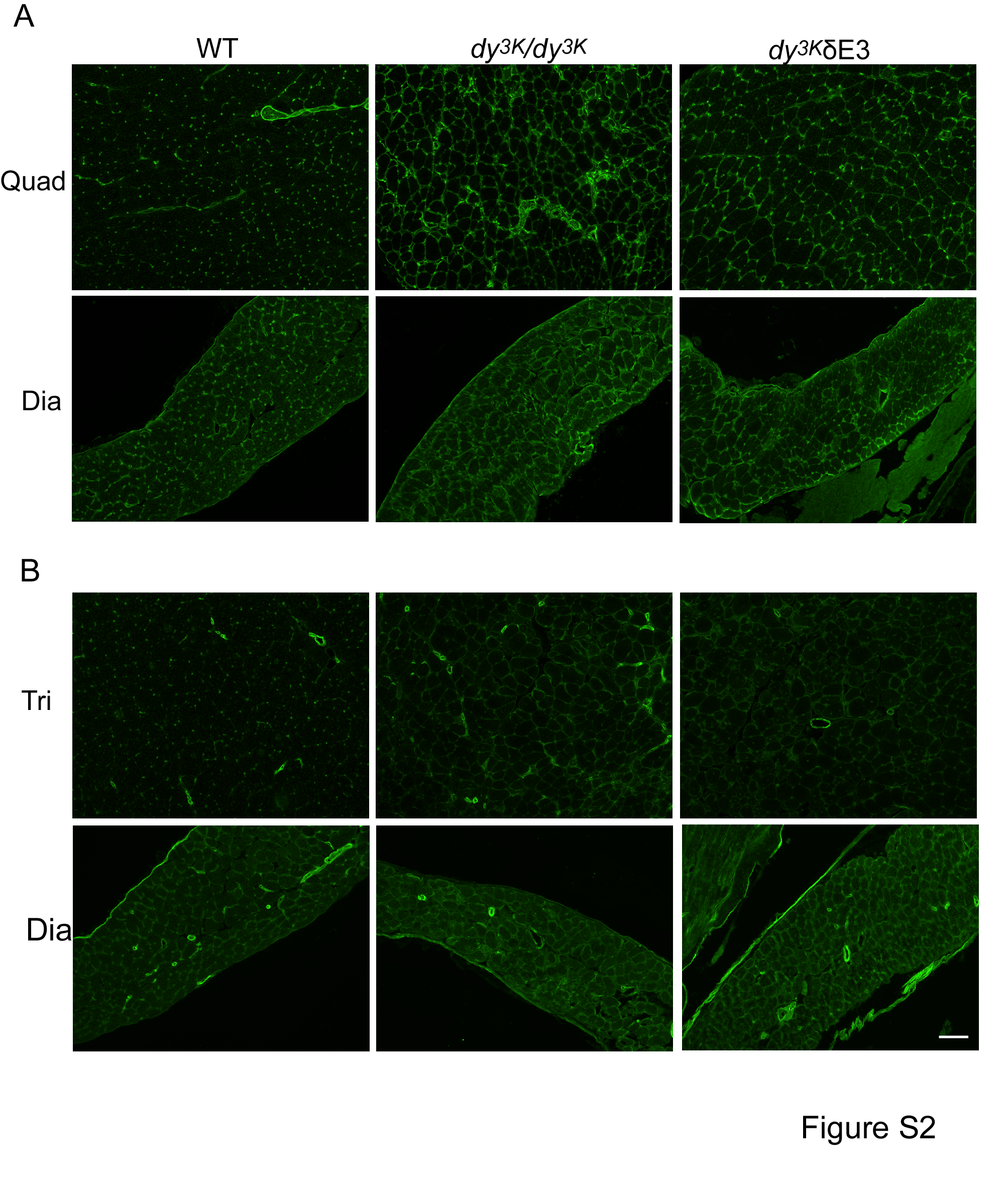

Supplement: Figure S2 — Immunostaining of LMα4 and α5 chains. Cross-sections of quadriceps femoris (Quad), triceps brachii (Tri) and diaphragm (Dia) from 6-week-old wild-type, dy3K/dy3K and dy3K/δE3 mice were stained with antibodies against LMα4 chain (A) and α5 chain (B), respectively. Expression of LMα4 and α5 chains is increased at the muscle basement area in dy3K/dy3K mice and remains increased in dy3K/δE3 muscles. Four dy3K/δE3 animals were analyzed. Bar, 50 µm. (3.67 MB TIF) [file pone.0011549.s002.tif]

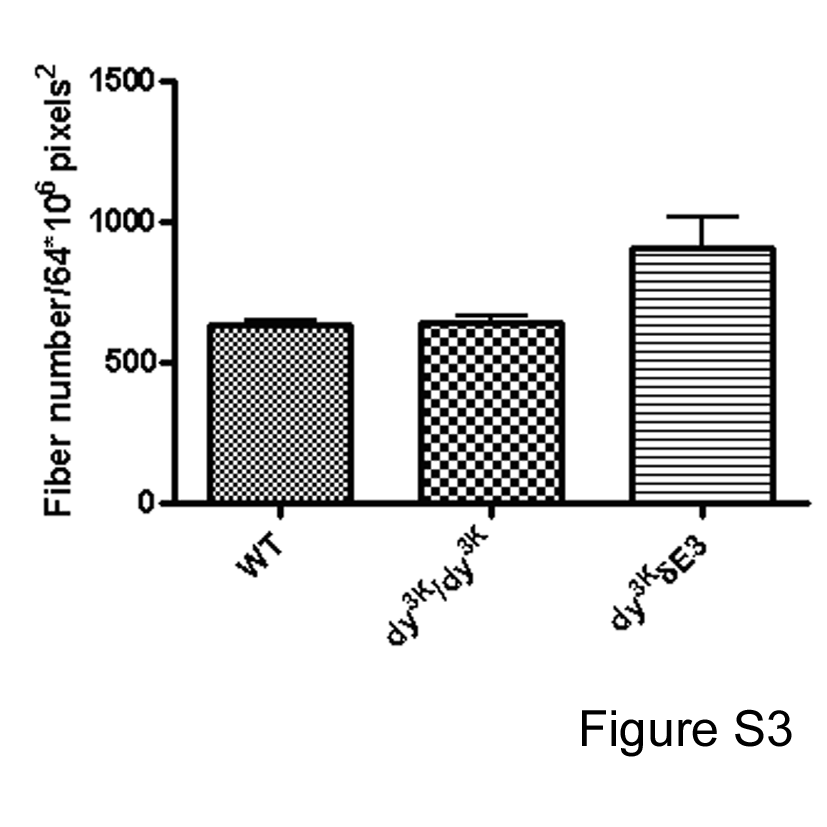

Supplement: Figure S3 — The numbers of fibers in a randomly selected area is not significantly different between the genotypes. (0.20 MB TIF) [file pone.0011549.s003.tif]
